# Somatic Kitl promotes mTOR to facilitate prophase I of meiosis in female embryonic gonads

**DOI:** 10.1038/s41419-025-08158-y

**Published:** 2025-11-17

**Authors:** Chang Liu, Ziyi Jin, Jiyu Chen, Jie Li, Guofeng Feng, Guoxing Yin, Yongqin Yu, Xiaoying Ye, Haowei Sun, Hua Zhang, Fei Gao, Lin Liu

**Affiliations:** 1 State Key Laboratory of Medicinal Chemical Biology, Nankai University, Tianjin, China; 2 Department of Cell Biology and Genetics, Nankai University, Tianjin, China; 3 State Key Laboratory of Agrobiotechnology, College of Biological Sciences, China Agricultural University, Beijing, China; 4 State Key Laboratory of Stem Cell and Reproductive Biology, Institute of Zoology, Chinese Academy of Sciences, Beijing, China

**Keywords:** Oogenesis, Extracellular signalling molecules

## Abstract

Homologous synapsis and recombination are the central events that take place in the prophase I of meiosis. Signaling that promotes the germ cell differentiation and prophase I remains elusive. Here we show a key Kitl/Kit signaling between somatic cells and germ cells in regulating meiotic prophase I in the mouse fetal gonad. Disruption of Kitl/Kit signaling, both in vivo and in vitro, impairs meiosis initiation, disrupts homologous synapsis and recombination. Moreover, mTOR/p-S6 signaling induced by Kitl/Kit elevates the levels of critical proteins such as Stra8, Sycp1 and Sycp3 for meiosis entry and homologous synapsis. Blocking Kitl/Kit signaling suppresses the mTOR and decreases the protein levels of Stra8, Sycp1, Sycp3 and Vasa, impairing the prophase I. In contrast, activating mTOR can rescue the meiotic defects caused by somatic *Kitl* deficiency. The activated p-AKT links Kitl/Kit to promoting mTOR/p-S6 signaling in the fetal germ cells. These findings reveal the critical functions and mechanisms of somatic Kitl in meiosis entry and homologous synapsis and recombination during the prophase I.

## Introduction

Germ cells undergo meiosis, a process of utmost significance in genetic development and the promotion of biological diversity. Any aberrations during meiotic division can result in infertility, premature ovarian failure, or congenital anomalies [1]. In the early stages of embryonic development, cells adjacent to the epiblast give rise to primordial germ cells (PGCs), which exhibit alkaline phosphatase (ALP) activity. Between embryonic days 7.5 and 10.5 (E7.5–E10.5), these PGCs proliferate, migrate through the hindgut, and ultimately settle in the genital ridge [2]. Unlike male PGCs, female PGCs enter meiosis at E12.5–E14.5 [3–6].

The traditional model posits that retinoic acid (RA) triggers female meiosis by activating the expression of stimulated by retinoic acid 8 (Stra8) [7–10]. However, this model remains controversial, as it relies primarily on studies involving the effects of exogenous all-trans retinoic acid (ATRA) on ex vivo gonadal cultures [11]. RA signaling is mediated by retinoic acid receptors (RARα, RARβ, RARγ) and retinoid X receptors (RXRs), with RA synthesized in the mesonephros and diffusing into the fetal ovary, while somatic ovarian cells also produce RA autonomously [12]. Aldehyde dehydrogenase 1A2 (ALDH1A2, or RALDH2) is the key enzyme for RA biosynthesis [13]. Paradoxically, RA signaling is dispensable for initiating meiosis in female germ cells in vivo, contradicting earlier models derived from exogenous RA exposure in cultured systems [14, 15].

Stra8, the only unequivocal “gatekeeper” of meiosis, is indispensable, as its knockout completely arrests meiotic progression in both sexes [11, 16]. Notably, the activation of key meiotic genes (e.g., *Rec8*, *Meioc*, *Sycp1*, *Spo11*) is dependent, but not completely, on Stra8 [17–21], indicating that potential additional regulatory signals in gonadal cells control meiosis-related gene expression. The transcription factor Meiosin cooperates with Stra8 to bind regulatory regions of meiotic genes and activate their expression [22, 23]. Other factors, including Dazl [17] and the Bmp2-Zglp1 pathway (via the modulation of H3K27me3 modifications at bivalent gene loci) [24], further regulate this process. As meiosis progresses, synaptonemal complex (SC) proteins, including Sycp1 and Sycp3, along with their associated components (Syce2, Syce3), ensure proper chromosome synapsis [25]. Moreover, multiple key proteins, including Rad51, Dmc1, Mlh1, Prdm9, Prc2, and Ythdc2, are essential for homologous chromosome synapsis, recombination and meiotic progression [26–29]. The timing of meiosis initiation is regulated by epigenetic factors such as the polycomb repressive complex PRC1, which promotes chromatin structural modifications and consequently controls the timing of Stra8 expression [30].

The precise regulation of these proteins is essential for the normal operation of meiosis. PGCs are instructed to enter meiosis by signaling molecules or signals produced by ovarian somatic cells [31–33]. Recent studies using in vitro models such as primordial germ cell-like cells (PGCLCs) and E12.5 PGCs have shown that the absence of gonadal somatic cells disrupts meiotic entry [34–36], further emphasizing the importance of direct somatic–germ cell interactions in meiosis. Key signals from the surrounding somatic cells to induce and complete meiosis remain to be explored. Our data revealed that Kitl from somatic cells facilitates meiosis entry and progression by increasing Stra8 protein levels and, consequently, other key meiosis-related proteins mediated by mTOR signaling.

## Results

### The Kitl/Kit signaling exhibits robust crosstalk between somatic cells and germ cells

**Fig. 1:**
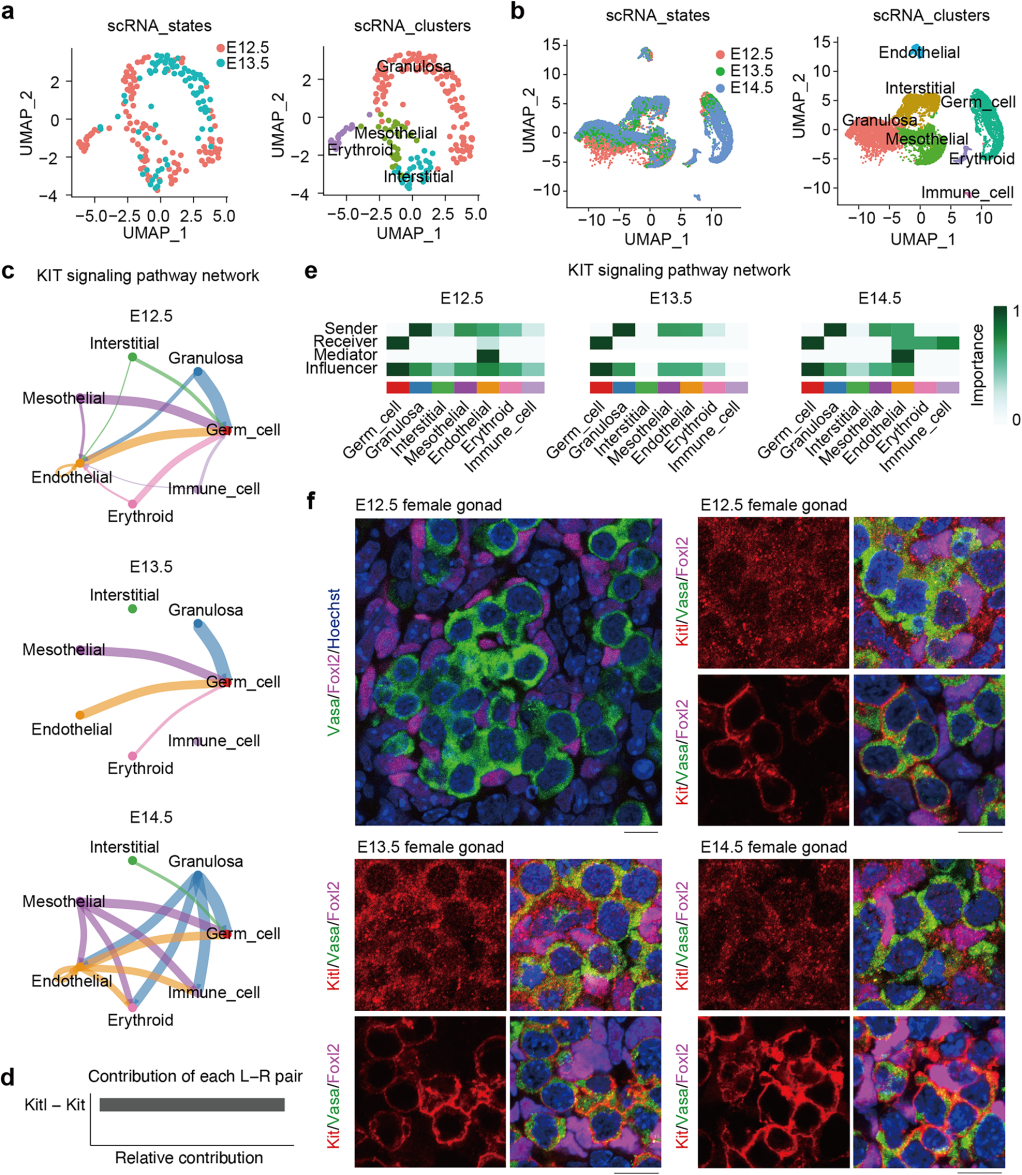
Interaction of Kitl/Kit signaling between germ cells and somatic cells in mouse embryonic gonads. **a** Two-dimensional (2D) visualization of clusters based on different samples (left) and transcriptional patterns (right) via UMAP from our dataset A: GSE181501 in embryonic (E12.5 and E13.5) gonads. **b** 2D visualization of clusters based on different samples (left) and transcriptional patterns (right) via UMAP from dataset B: GSE128553 in E12.5, E13.5 and E14.5 gonads [40]. **c** KIT signaling pathway network in E12.5, E13.5 and E14.5 female gonads, based on dataset B [40]. The thickness of the lines indicates the strength of the relationship. **d** Relative contribution of ligand–receptor pairs to the overall communication network of KIT signaling pathways between germ cells and somatic cells. **e** Heatmap shows the role (sender secretes ligands, receiver expresses receptor, mediator modulates signal transduction, and influencer orchestrates global communication) of each cell group in the KIT signaling network, based on dataset B [40]. **f** Immunofluorescence of Kitl, Foxl2 and Vasa (top) and Kit, Foxl2 and Vasa (bottom) in female gonad sections from E12.5, E13.5 and E14.5 fetus. Magenta, Foxl2, indicative of pregranulosa or granulosa cells; red, Kitl or Kit; green, Vasa; blue, nuclei counterstained with Hoechst 33342. Scale bar: 20 μm.

To investigate the potential role of gonadal somatic cells in regulating the initiation and progression of germ cell meiosis, we performed single-cell RNA-seq analysis at E12.5 and E13.5 [37]. Through UMAP visualization and clustering analysis, we successfully identified four distinct clusters corresponding to granulosa cell (*Wnt6*^+^), mesothelial cell (*Upk3b*^+^), endothelial cell (*Col1a1*^+^) and erythroid cell (*Alas2*^+^) types on the basis of established marker genes (Fig. 1a and Supplementary Fig. S1a) [38]. Each stage was subsequently isolated separately, and CellChat was used to identify the signaling pathways associated with somatic cells [39]. Our findings revealed that the WNT, BMP, and KIT signaling pathways were expressed in gonadal somatic cells at the E12.5 stage, whereas the KIT, ACTIVIN, and IGF signaling pathways were expressed in gonadal somatic cells at the E13.5 stage (Supplementary Fig. S1b).

Furthermore, we explored the signaling pathways involved in the interaction between somatic cells and germ cells at three distinct developmental stages of female gonads, E12.5, E13.5, and E14.5, via CellChat. Two independent 10× single-cell RNA-seq datasets from Ge W et al. and Niu W et al. were used for CellChat analysis [40, 41]. After implementing rigorous quality control measures and adjusting for batch effects, we employed UMAP visualization to identify seven distinct clusters corresponding to different time periods [42]. The differentially expressed genes were subsequently determined for each cluster on the basis of cell identity annotation and known marker genes [40]. The gonads at E12.5, E13.5, and E14.5 were categorized into seven distinct populations (Fig. 1b): granulosa cells, mesothelial cells, interstitial cells, germ cells, endothelial cells, erythroid cells and immune cells. We subsequently extracted each period separately and employed CellChat to identify the overall degree of intercellular communication between somatic cells and germ cells (Supplementary Fig. S1c). Notably, the most prominent signaling pathways in E12.5, E13.5 and E14.5 germ cells were KIT and WNT.

On the basis of our analysis of Smart-Seq2 data combined with insights from CellChat analysis of 10× single-cell data, the presence of a significant KIT, ACTIVIN, and BMP signaling pathway may exert a pivotal influence on meiotic initiation and progression. The BMP signaling pathway is involved in the interaction between somatic cells and germ cells at E12.5 and E13.5 (Supplementary Fig. S1d) but has a diminished effect at E14.5, implying its crucial involvement in meiotic initiation, as previously reported by other studies [43]. Furthermore, the germ cells that highly expressed KIT at E12.5, E13.5, and E14.5 interacted mainly with granulosa cells and mesothelial and endothelial cells (Fig. 1c).

Through quantitative comparison, CellChat assists in identifying differentially expressed ligands and receptors within each cell subpopulation [39], revealing a significant interaction between the *Kit* receptor and *Kitl* signal during the E12.5–E14.5 stage (Fig. 1d). While the *Kitl* signal is strongly produced by granulosa cells, it can also be found in other somatic cell types, such as mesothelial and endothelial cells (Fig. 1e). PGCs at E12.5, E13.5, and E14.5 expressed *Kit* signaling and received *Kitl* produced from somatic cells (Fig. 1e). Similar expression patterns of Kitl/Kit in female gonads can also be confirmed by analyzing other 10× single-cell RNA-seq data (Supplementary Fig. S1e–g) [41]. Moreover, immunofluorescence indicated that Kit was expressed in Vasa^+^ germ cells, whereas Kitl was expressed in or around the somatic cells and was notably Foxl2 positive, indicative of granulosa cells surrounding the germ cells in female gonads at the E12.5, E13.5 and E14.5 stages (Fig. 1f). However, it is unknown whether Kitl/Kit signaling may function in the initiation and progression of meiosis at these stages.

### *Kitl* deficiency impairs the occurrence of meiosis and homologous synapsis and recombination

To elucidate the potential impact of the Kitl/Kit signaling pathway on the entry and progression of germ cell meiosis, we attempted to knock out *Kitl* in somatic cells and evaluate meiosis in germ cells in a mouse model. Given that granulosa cells are the major producers of Kitl (Fig. 1e, f and Supplementary Fig. S1g), we generated granulosa cell-specific conditional knockout mice for *Kitl* via the *Kitl*^flox/flox^ [44] and *Foxl2*-Cre [45] mouse models (Supplementary Fig. S2a). Gonads from E12.5, E13.5, E14.5 and E16.5 embryos were isolated for subsequent analysis. Genotyping of female mouse gonads was performed via PCR to confirm the *Kitl* gene knockout (Supplementary Fig. S2b). However, there was no significant disparity in embryo size or gonad dimensions between *Kitl*^flox/+^; *Foxl2*-cre (*Kitl* f/+ cre) and *Kitl*^flox/flox^; *Foxl2*-cre (*Kitl* f/f cre, or *Kitl* cKO), regardless of the meiotic stage (Supplementary Fig. S2c). Immunofluorescence analysis further confirmed that Kitl protein was absent or present at minimal levels in and around *Kitl* cKO granulosa cells (Supplementary Fig. S2d).

**Fig. 2:**
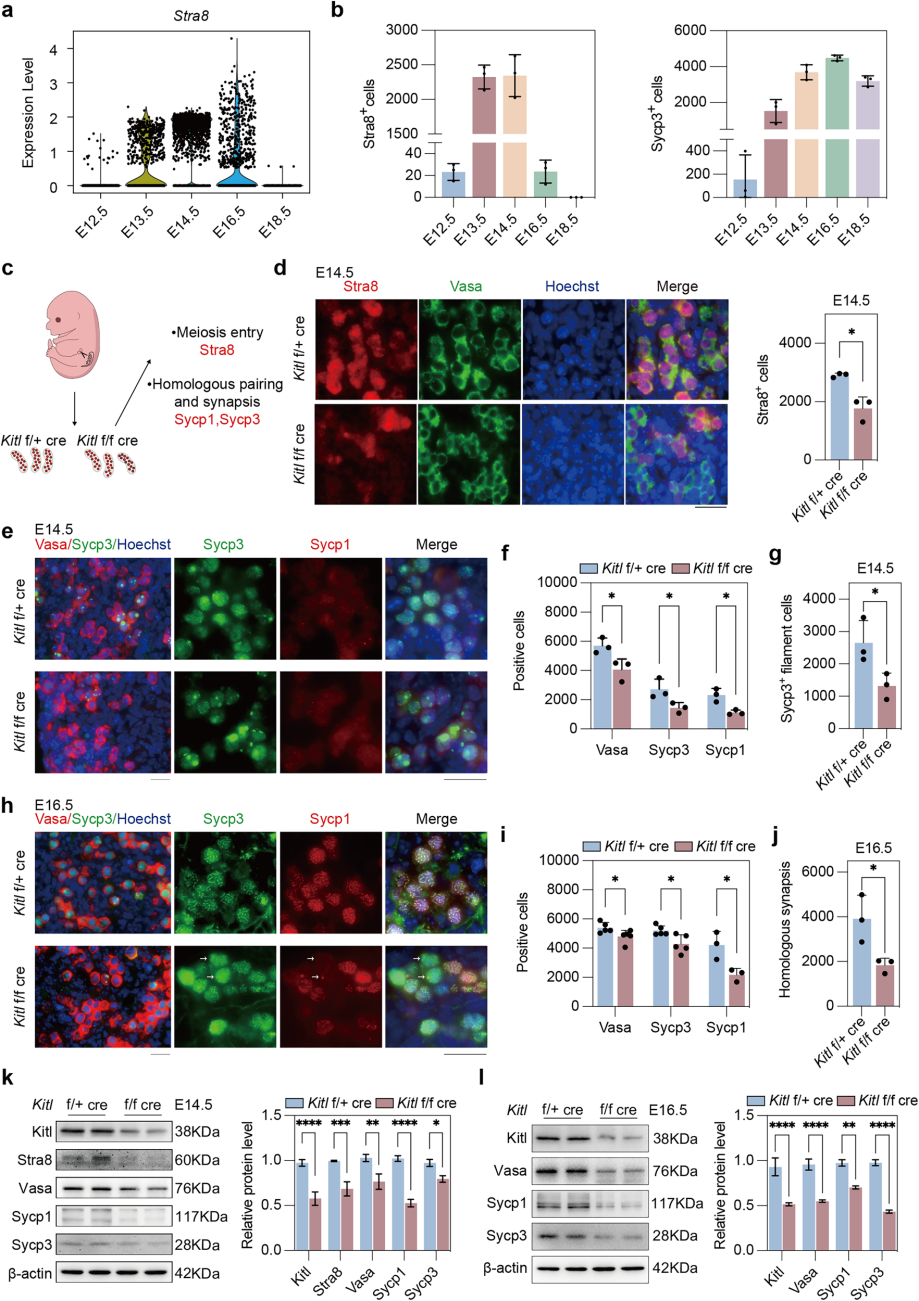
*Kitl* deficiency in somatic granulosa cells decreases meiosis entry and key proteins for meiosis. **a** Violin plot showing *Stra8* RNA levels by scRNA-seq of female gonads at various embryonic timepoints (E12.5–E18.5) from dataset B [40] and dataset C: GSE136441 [41]. **b** The number of Stra8^+^ and Sycp3^+^ cells per gonad at various embryonic timepoints (E12.5–E18.5) from ICR female mouse gonads. *n* = 3 fetal mouse gonads. **c** Experimental design involving *Kitl* f/f cre and f/+ cre female fetal gonads and examination of key meiosis markers. **d** Immunofluorescence and quantification of Vasa and Stra8 per gonad in E14.5 *Kitl* f/f cre and f/+ cre female mouse gonads. Green, Vasa; red, Stra8, marker for meiosis entry; blue, nuclei counterstained with Hoechst 33342. Scale bar: 20 μm. *n* = 3 gonads. **e** Representative immunofluorescence images of Vasa and Sycp3 (left) or Sycp1 and Sycp3 (right) in E14.5 female gonads from *Kitl* f/f cre and f/+ cre mice. Green, Sycp3; red, Vasa or Sycp1; blue, nuclei counterstained with Hoechst 33342. Scale bar: 20 μm. **f** The number of Vasa^+^, Sycp3^+^ and Sycp1^+^ cells per gonad in E14.5 *Kitl* f/f cre and f/+ cre female mouse gonads. *n* = 3 gonads. **g** The number of Sycp3^+^ filament cells per gonad in E14.5 *Kitl* f/f cre and f/+ cre female mouse gonads. *n* = 3 gonads. **h** Immunofluorescence of Vasa and Sycp3 (left) or Sycp1 and Sycp3 (right) in E16.5 female gonads from *Kitl* f/f cre and f/+ cre mice. The arrows indicate the germ cells expressing Sycp3 but not Sycp1. Scale bar: 20 μm. **i** The number of Vasa^+^, Sycp3^+^ and Sycp1^+^ cells per gonad in E16.5 *Kitl* f/f cre and f/+ cre female mouse fetus. *n* = 3 gonads. **j** Quantification of the number of cells with homologous synapsis per gonad, as evidenced by coimmunofluorescence of Sycp1 and Sycp3 in E16.5 *Kitl* f/f cre and f/+ cre female gonads. *n* = 3 gonads. **k** Western blot and quantification of Kitl, Stra8, Vasa, Sycp1 and Sycp3 protein levels in E14.5 *Kitl* f/f cre and f/+ cre female gonads. β-actin served as a loading control. *n* = 2 independent experiments. **l** Western blot and quantification of Kitl, Vasa, Sycp1 and Sycp3 protein levels in E16.5 *Kitl* f/f cre and f/+ cre female gonads. β-actin served as a loading control. *n* = 2 independent experiments. The data are presented as the means ± SDs. *p < 0.05; **p < 0.01; ***p < 0.001; ****p < 0.0001.

Analysis of 10x single-cell RNA-seq data revealed that *Stra8* was minimally expressed at E12.5, significantly increased at E13.5, reached its peak at E14.5, and was nearly undetectable at E16.5 and E18.5 (Fig. 2a and Supplementary Fig. S2e). Quantitative analysis of frozen tissue sections from gonads from ICR mice at different stages confirmed the high expression of Stra8 between E13.5 and E14.5 (Fig. 2b and Supplementary Fig. S2f). Additionally, we examined the expression of Sycp3, which was highest at E16.5 (Fig. 2b). Therefore, we focused on analyzing phenotypic changes at E14.5 and E16.5 to gain a comprehensive understanding of the impact of *Kitl* deficiency.

We initially evaluated the impact of *Kitl* deficiency in granulosa cells on germ cell viability and meiosis initiation in female mouse gonads via immunofluorescence staining for the germ cell marker Vasa, the meiosis entry marker Stra8, and the SC proteins Sycp3 and Sycp1 (Fig. 2c). Stra8 staining of gonad sections revealed a notable decrease in the number of Stra8^+^ cells in E14.5 *Kitl* f/f cre mice compared with those in *Kitl* f/+ cre mice (Fig. 2d). In E14.5 *Kitl* f/f cre mice, a significant decrease in the number of Vasa-positive cells was detected (Fig. 2e, f), as well as Sycp3^+^ and Sycp1^+^ germ cells (Fig. 2f), indicating a reduction in both total germ cell counts and the proportion of germ cells that entered meiosis with *Kitl* deficiency in granulosa cells. In addition, we were surprised to find that the number of filaments of Sycp3^+^ germ cells significantly decreased with *Kitl* deficiency in granulosa cells at E14.5 (Fig. 2g). In the female gonads at E16.5, a modest reduction in Vasa^+^ germ cell expression was observed (Fig. 2h, i), albeit less pronounced than that at E14.5. Concurrently, the number of Sycp3^+^ and Sycp1^+^ meiocytes also decreased when *Kitl* was deficient in granulosa cells (Fig. 2i). Consequently, synapsis in meiocytes, as shown by the staining of Sycp1 and Sycp3, was reduced without *Kitl* in granulosa cells (Fig. 2j), indicating that Kitl/Kit signaling is important for normal synapsis. To determine whether loss of germ cells in *Kitl* cKO resulted from apoptosis or meiotic failure, we performed TUNEL and cleaved caspase-3 staining in E14.5 and E16.5 gonads (Supplementary Fig. S3a), and showed consistently low numbers of apoptotic cells across all stages and genotypes, with minimal differences between *Kitl* f/+ cre and *Kitl* f/f cre mice (Supplementary Fig. S3b). These results indicate that germ cells reduction in the *Kitl* cKO is unlikely to be caused by increased apoptosis. It remains to be determined whether the meiotic failure contributes to the loss of germ cells. Moreover, the Vasa, Sycp1, and Sycp3 protein levels revealed by immunofluorescence microscopy were corroborated by western blot analysis (Fig. 2k, l). In short, Kitl/Kit signaling is critical for meiotic initiation and progression.

**Fig. 3:**
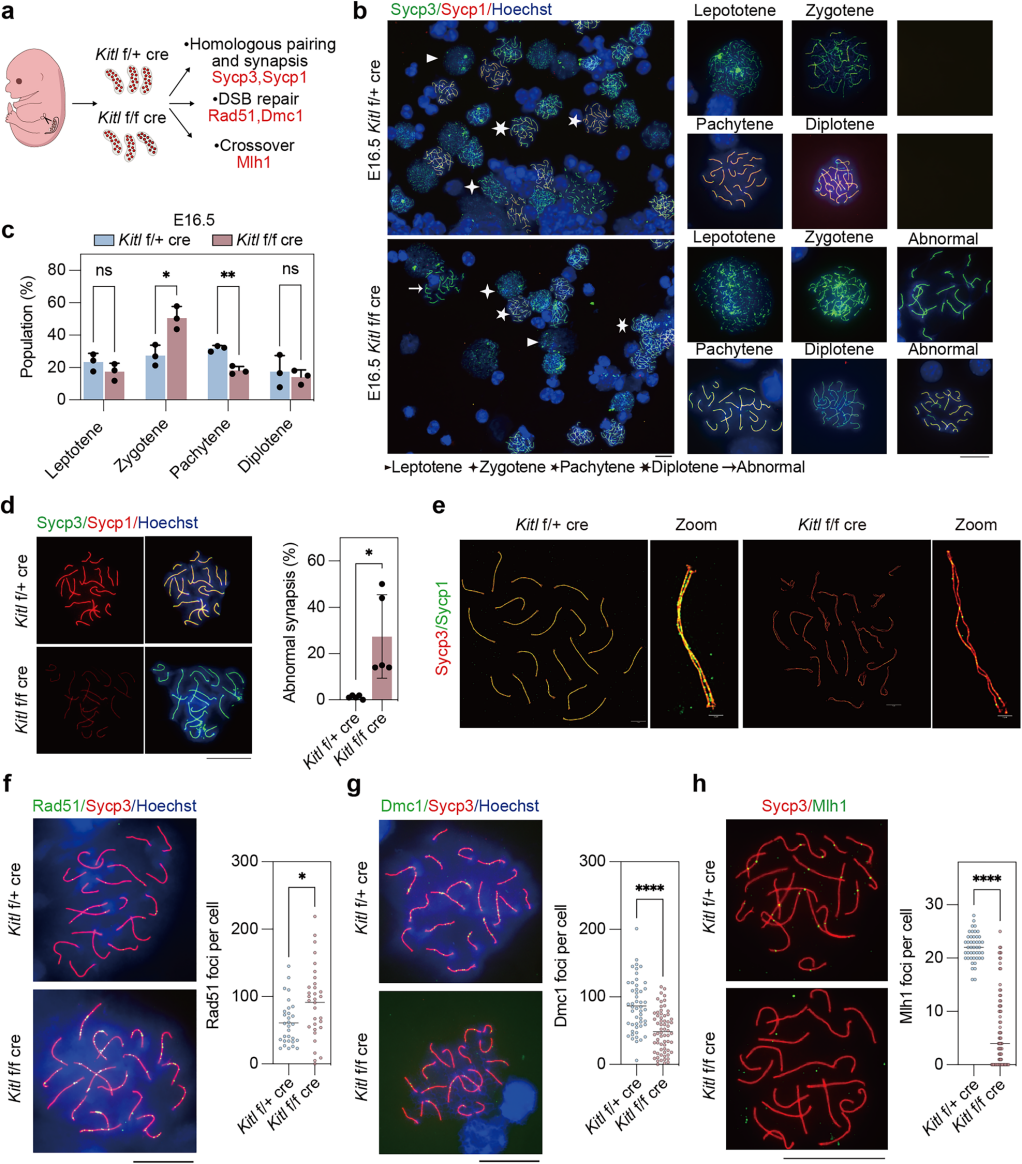
*Kitl* deficiency impairs homologous synapsis and recombination. **a** Experimental design involving E16.5 *Kitl* f/f cre (cKO) and f/+ cre (WT) female gonads and examination of key meiosis markers. **b** Representative immunofluorescence images of chromosome spreads from E16.5 *Kitl* f/f cre and f/+ cre female gonads stained for Sycp3 and Sycp1. Green, Sycp3; red, Sycp1; blue, nuclei. Scale bar: 20 μm. **c** Statistical counts showing the percentage of meiocytes at various stages as indicated in (**b**). *n* = 3 independent experiments. **d** Immunofluorescence of Sycp3 and Sycp1 in E16.5 *Kitl* f/f cre and f/+ cre female gonads, and the percentages of abnormally paired pachytene germ cells. *n* = 5 independent experiments. Scale bar: 20 μm. **e** Representative immunofluorescence images of Sycp3 and Sycp1 in E16.5 *Kitl* f/f cre and f/+ cre female gonads photographed by Stedycon. Green, Sycp1; red, Sycp3. Scale bar: 5 μm for full, 1 μm for zoom. **f** Immunofluorescence of Rad51 and Sycp3 in chromosome spreads of E16.5 *Kitl* f/f cre and f/+ cre female gonads and quantification of the number of Rad51 foci per nucleus. Green, Rad51; red, Sycp3; blue, nuclei counterstained with Hoechst 33342; *n* ≥ 30 nuclei for each group. Scale bar: 20 μm. **g** Immunofluorescence of Dmc1 and Sycp3 in chromosome spreads of E16.5 *Kitl* f/f cre and f/+ cre female gonads and quantification of the number of Dmc1 foci per nucleus. *n* ≥ 50 nuclei for each group. Scale bar: 20 μm. **h** Immunofluorescence of Mlh1 and Sycp3 in chromosome spreads of E16.5 *Kitl* f/f cre and f/+ cre female gonads and quantification of the number of Mlh1 foci per nucleus. *n* ≥ 45 nuclei for each group. Scale bar: 20 μm. The data are presented as the means ± SDs. n.s. not significant, *p* > 0.05; *p < 0.05; **p < 0.01; ****p < 0.0001.

To investigate the impact of *Kitl* deficiency on critical meiotic events, particularly homologous synapsis during prophase I, we conducted an in-depth analysis of female gonads at E16.5 (Fig. 3a). Immunofluorescence of surface chromosome spreads was used to assess the chromosomal configuration [46] of *Kitl* f/f cre and f/+ cre mice at the E16.5 stage (Fig. 3b). Statistical analysis of germ cells at various meiotic stages revealed that the majority of germ cells in *Kitl* f/f cre mice were arrested at the zygotene stage, in contrast to those in *Kitl* f/+ cre mice (Fig. 3c). While some germ cells in *Kitl* f/f cre mice proceeded through meiosis, a subset exhibited abnormalities with notably reduced Sycp1 protein filaments (Fig. 3b: abnormal, Fig. 3d). To better investigate the chromosomal synapsis status in *Kitl* f/f cre and f/+ cre mice, higher-resolution StedyCon microscopy was utilized, revealing significantly reduced Sycp1 filaments, as well as less tight synapsis, in *Kitl* f/f cre mice during meiosis prophase I (Fig. 3e).

Abnormalities in synapsis are typically associated with defects in homologous recombination and double-strand break (DSB) repair [47]. To assess the impact of *Kitl* deficiency on these processes, we examined the expression patterns of key homologous recombination proteins, Rad51 and Dmc1, during the pachytene phase. Coimmunofluorescence of Rad51, Dmc1, and Sycp3 revealed an increase in the Rad51 signal (Fig. 3f) but a decrease in the Dmc1 signal (Fig. 3g). Thus, the synapsis defects could result in the DSB repair deficiency without *Kitl*. DSB repair failure ultimately hampers crossover, as evidenced by a significant reduction in the number of Mlh1 foci, a critical marker for crossover events, in *Kitl*-deficient mice (Fig. 3h). In summary, disruption of *Kitl* in granulosa cells results in a reduced number of germ cells entering meiosis, accompanied by defects in synapsis, DSB repair, and crossover formation during meiosis.

### Kitl/Kit signaling regulates the transcription of key genes for meiosis initiation and progression

**Fig. 4:**
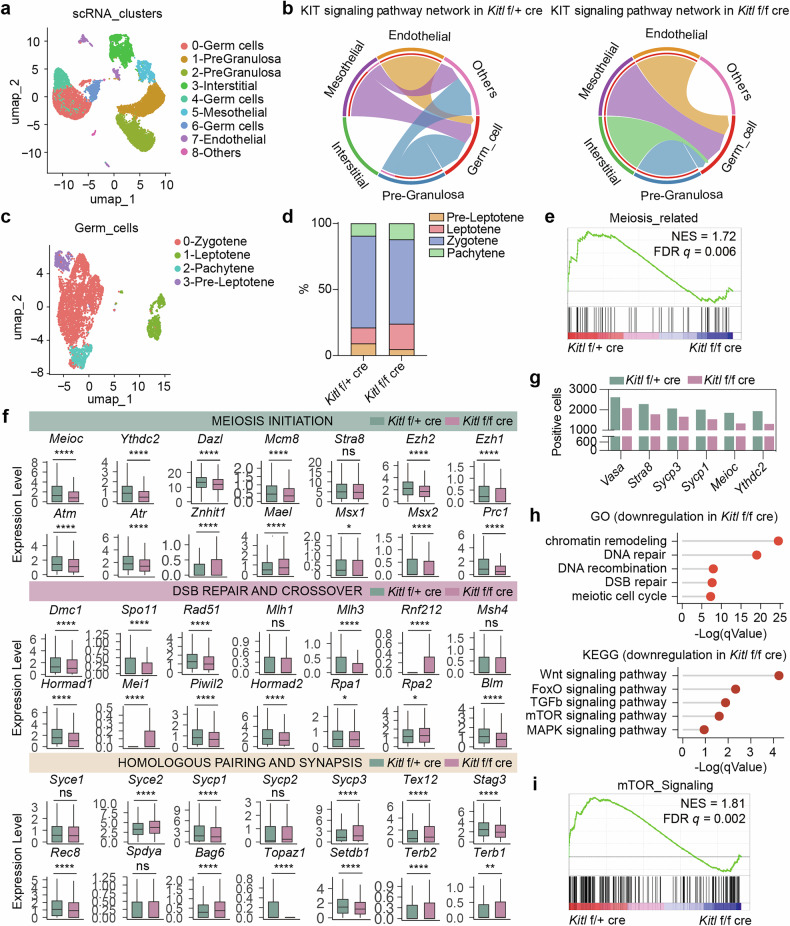
*Kitl* deficiency alters the transcriptome of germ cells. **a** 2D visualization of clusters on the basis of transcriptional patterns of E14.5 *Kitl* f/f cre (cKO) and f/+ cre (WT) gonads via UMAP. **b** Interaction of the KIT signaling pathway in *Kitl* f/f cre and f/+ cre gonads. **c** 2D visualization of clusters on the basis of transcriptional patterns of *Kitl* f/f cre and f/+ cre germ cells via UMAP. **d** Percentage of germ cells at various stages, including preleptotene, leptotene, zygotene, and pachytene in *Kitl* f/f cre and f/+ cre gonads. **e** GSEA of meiosis-related genes in *Kitl* f/f cre and f/+ cre germ cells. **f** Bar plot showing differentially expressed genes for meiosis in *Kitl* f/f cre and f/+ cre germ cells. **g** The number of germ cells expressing meiotic genes, *Vasa*, *Stra8*, *Sycp3*, *Sycp1*, *Meioc*, and *Ythdc2*. **h** Downregulated genes and signaling pathways in *Kitl* f/f cre germ cells, compared with those of *Kitl* f/+ cre germ cells. **i** GSEA of the mTOR signaling pathway in *Kitl* f/f cre and f/+ cre germ cells.

To investigate the potential molecular basis by which Kitl signaling in somatic cells affects meiotic progression in germ cells, we performed single-cell RNA-seq to analyze the transcriptome of female gonads at E14.5, a stage when most germ cells enter the zygotene phase [48]. We examined the RNA profiles of *Kitl* f/f cre and f/+ cre female gonads via 10× single-cell RNA sequencing. Following rigorous data quality control, a total of 14,645 cells were analyzed, including 7839 *Kitl* f/+ cre cells and 6806 *Kitl* f/f cre cells. Cluster analysis was performed via Seurat, which is based on UMAP dimensionality reduction (Fig. 4a). Distinct cell populations, including germ cells (*Dazl*, *Ddx4*, and *Cenpf*), pregranulosa cells (*Wnt6*), mesothelial cells (*Lhx9*), endothelial cells (*Pecam1*) and interstitial cells (*Col1a1*), were identified via specific markers (Supplementary Fig. S4a) [40]. The proportion of germ cells decreased, whereas the proportion of granulosa cells increased in the gonads of *Kitl* f/f cre females, as shown by the cell population ratio (Supplementary Fig. S4b). FeaturePlot analysis confirmed the absence of *Kitl* expression in the *Foxl2*^+^ granulosa cells of the *Kitl* f/f cre mice (Supplementary Fig. S4c).

To further explore intercellular interactions, we utilized CellChat and identified a significant reduction in Kit signaling, which mediates communication between granulosa and germ cells, in *Kitl* f/f cre gonads compared with those of *Kitl* f/+ cre controls (Fig. 4b). GO and KEGG analyses of granulosa cells revealed significant downregulation of GPCR signaling and cell‒cell adhesion pathways following *Kitl* deletion, suggesting that granulosa cells may affect germ cells through these mechanisms (Supplementary Fig. S4d).

We then isolated and re-clustered the germ cells to further investigate the effects of *Kitl* deficiency on somatic cell signaling in germ cells (Fig. 4c). Germ cell populations at the preleptotene, leptotene, zygotene, and pachytene stages were identified on the basis of the expression of meiotic markers (Supplementary Fig. S4e) [41]. *Kitl* deficiency led to an abnormal distribution of germ cells at different stages across meiosis (Fig. 4d). Differential gene expression analysis between *Kitl* f/f cre and f/+ cre germ cells revealed 1960 downregulated genes and 706 upregulated genes in the *Kitl* f/f cre germ cells compared to *Kitl* f/+ cre (Supplementary Fig. S4f). Gene set enrichment analysis (GSEA) revealed a significant decrease in meiosis-related pathways after *Kitl* deficiency (Fig. 4e). Key genes for meiotic initiation, such as *Meioc* [21], *Ythdc2* [49], and other genes for homologous recombination and pairing, such as *Spo11* [50], *Hormad1* [51], and *Brca2*, were also significantly downregulated without *Kitl* (Fig. 4f). qPCR validation of *Meioc*, *Ythdc2*, and *Spo11* confirmed the downregulation of these genes with *Kitl* deficiency, which was consistent with the RNA sequencing data (Supplementary Fig. S4g). Additionally, we assessed the number of cells expressing meiosis-related genes (*Vasa*, *Stra8*, *Sycp3*, *Sycp1*, *Meioc*, and *Ythdc2*) and found that although *Stra8* expression at the mRNA level did not significantly change, the number of cells expressing *Stra8* was reduced in *Kitl* f/f cre gonads (Fig. 4g).

GO and KEGG analyses revealed significant downregulation of DNA repair processes and chromatin organization during the meiotic cell cycle in *Kitl* cKO gonads (Fig. 4h). Additionally, the Wnt, FoxO, and mTOR signaling pathways were significantly downregulated without *Kitl* (Fig. 4h), suggesting that abnormal meiotic progression may occur through these pathways in *Kitl* cKO mice. Western blot analysis confirmed that *Kitl* deficiency decreased the p-AKT/AKT, Foxo3a, and p-Stat3 signaling pathways but not the p-Erk/Erk signaling pathway in germ cells (Supplementary Fig. S4h). GSEA further demonstrated the widespread reduction in signaling pathway activity, with the mTOR pathway exhibiting the most significant changes (Fig. 4i and Supplementary Fig. S4i).

### Inhibition of Kit suppresses the initiation and progression of meiosis

**Fig. 5:**
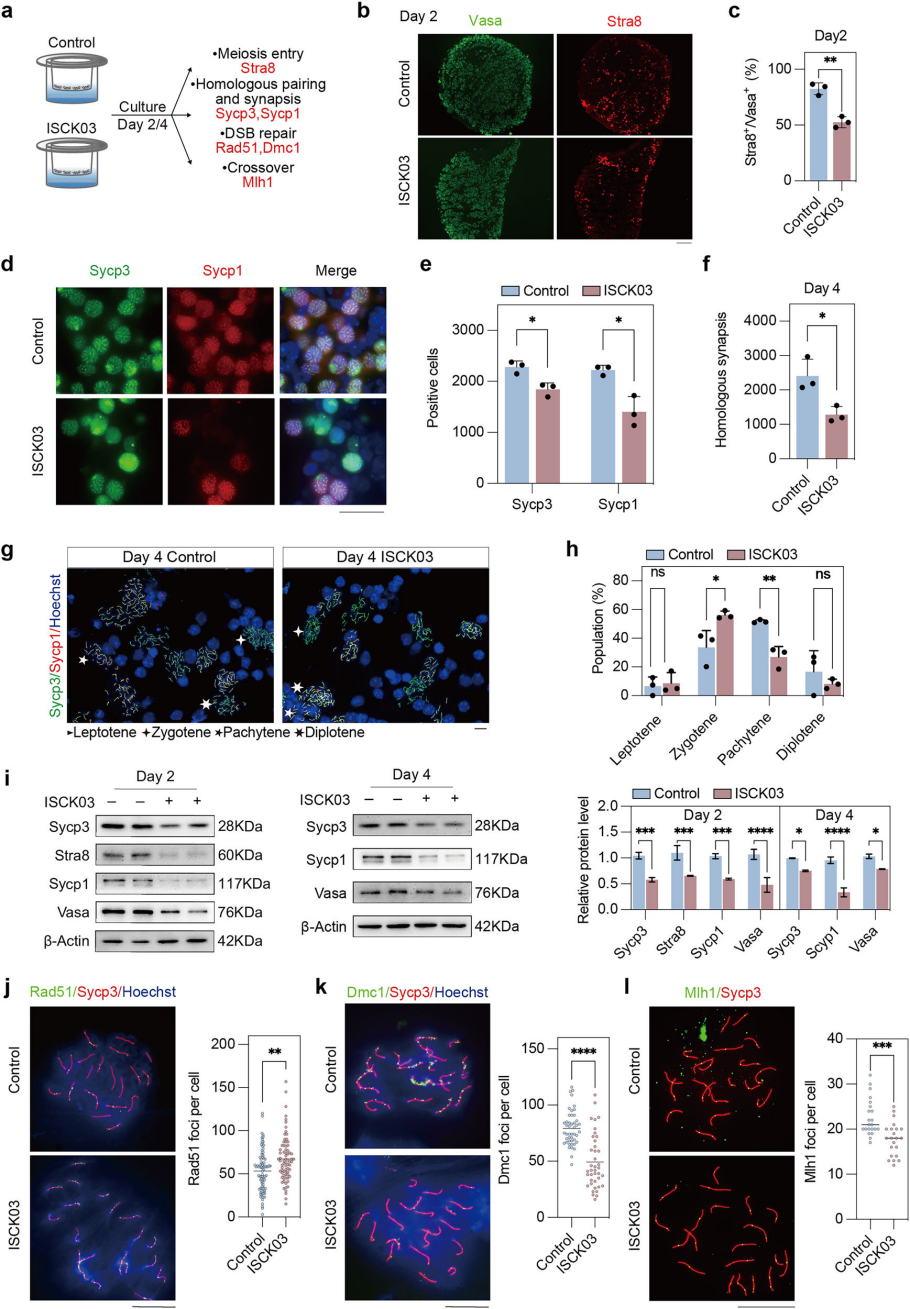
Inhibition of Kit decreases meiosis entry and homologous recombination. **a** Experimental design involving E12.5 female gonads treated with ISCK03 (Kit inhibitor) or without ISCK03 (control) for 2 or 4 days and examined for key meiosis markers. **b** Representative immunofluorescence images of Vasa and Stra8 in control and ISCK03-treated gonads cultured for 2 days. Green, Vasa; red, Stra8. Scale bar: 100 μm. **c** Quantification of the percentage of Stra8^+^ cells/Vasa^+^ cells in control and ISCK03-treated gonads on day 2. *n* = 3 fetal mouse gonads. **d** Representative immunofluorescence images of Sycp1 and Sycp3 in control and ISCK03-treated gonads cultured for 4 days. Green, Sycp3; red, Sycp1; blue, nuclei counterstained with Hoechst 33342. Scale bar: 20 μm. **e** The number of Sycp3^+^ and Sycp1^+^ cells per gonad in control and ISCK03-treated gonads cultured for 4 days. *n* = 3 gonads. **f** Quantification of the number of cells with homologous synapsis per gonad, as evidenced by co-immunofluorescence of Sycp1 and Sycp3 in control and ISCK03-treated gonads cultured for 4 days. *n* = 3 fetal mouse gonads. **g** Representative immunofluorescence images of surface chromosome spreads of germ cells from the control and ISCK03-treated gonads on day 4 stained for Sycp3 and Sycp1. Green, Sycp3; red, Sycp1; blue, nuclei counterstained with Hoechst 33342. Scale bar: 20 μm. **h** Statistical counts showing the percentage of meiocytes on day 4 at various stages as indicated. *n* = 3 independent experiments. **i** Western blot and quantification of the Stra8, Sycp3, Sycp1 and Vasa protein levels in control and ISCK03-treated gonads on days 2 and 4 of culture. β-actin was used as a loading control. *n* = 2 independent experiments. **j** Immunofluorescence of Rad51 and Sycp3 in chromosome spreads of cultured day 4 control and ISCK03-treated gonads and quantification of the number of Rad51 foci per nucleus. *n* ≥ 75 nuclei for each group. Scale bar: 20 μm. **k** Immunofluorescence of Dmc1 and Sycp3 in chromosome spreads of control and ISCK03-treated gonads cultured for 4 days and quantification of the number of Dmc1 foci per nucleus. *n* ≥ 40 nuclei for each group. Scale bar: 20 μm. **l** Immunofluorescence of Mlh1 and Sycp3 in chromosome spreads of cultured day 4 control and ISCK03-treated gonads and quantification of the number of Mlh1 foci per nucleus. *n* ≥ 22 nuclei for each group. Scale bar: 20 μm. The data are presented as the means ± SDs. n.s., not significant, *p* > 0.05; *p < 0.05; **p < 0.01; ***p < 0.001; ****p < 0.0001.

We also employed an in vitro culture system to examine the effect of Kit inhibition on meiotic progression. E12.5 gonads were cultured for 2 or 4 days with or without the Kit inhibitor ISCK03 [52], and the expression of meiosis-related markers was assessed at different stages (Fig. 5a). Immunofluorescence microscopy of the Vasa and Stra8 proteins in gonad sections cultured for 2 days revealed a significant reduction in the proportion of germ cells expressing the meiotic initiation marker Stra8 following ISCK03 treatment (Fig. 5b, c). Additionally, immunofluorescence of Sycp3 and Sycp1 revealed a significantly lower number of Sycp3^+^ and Sycp1^+^ cells in the ISCK03-treated gonads than in the control gonads on day 4 (Fig. 5d, e).

Moreover, the number of meiocytes that underwent normal synapsis, as evidenced by co-immunofluorescence of Sycp3 and Sycp1, was reduced in ISCK03-treated gonads (Fig. 5f). We performed immunofluorescence analysis of chromosome spreads following 4 days of in vitro culture with ISCK03 and compared the results with those of the controls (Fig. 5g). When meiosis predominantly reached the pachytene stage in the control, ISCK03 reduced the proportion of germ cells transitioning into pachytene, whereas a greater proportion remained at the zygotene stage (Fig. 5h). ISCK03 caused more severe defects in meiosis than did *Kitl* cKO gonads, likely suggesting that *Kitl* cKO depleted Kitl mostly in granulosa cells, whereas ISCK03 inhibited Kit signaling in germ cells, regardless of the source of Kitl. Western blotting and ImageJ [53] analysis further confirmed that the protein levels of Stra8, Sycp1, Sycp3, and Vasa were decreased by the inhibition of Kit (Fig. 5i).

Notably, an increase in the number of Rad51 foci (Fig. 5j), a decrease in the number of Dmc1 foci (Fig. 5k), and a reduction in the number of Mlh1 foci (Fig. 5l) were detected in the ISCK03-treated gonads compared with the control gonads, suggesting that inhibition of Kit disrupts chromosomal DSB repair and the crossover formation process.

### mTOR signaling is involved in Kitl/Kit regulation of meiosis

To further investigate the underlying mechanisms of the Kitl/Kit signaling pathway in meiosis, we investigated the potential functions of the mTOR signaling pathways obtained from the aforementioned single-cell results (Fig. 4i). We hypothesized that the Kitl/Kit signaling pathway may modulate meiosis through its influence on the mTOR pathway.

To test this hypothesis, we first investigated the impact of the mTOR signaling pathway on meiotic entry, synapsis, DSB repair, and crossover formation by introducing rapamycin, an mTOR inhibitor [54], into gonads cultured in vitro (Supplementary Fig. S5a). Rapamycin suppressed meiotic entry, as evidenced by a reduction in the proportion of Stra8^+^/Vasa^+^ cells of rapamycin-treated gonads on day 2 (Supplementary Fig. S5b, c), indicating that inhibition of the mTOR pathway delays meiotic entry. Similarly, immunostaining of Sycp1 and Sycp3 (Supplementary Fig. S5d) revealed a decrease in the proportion of Sycp3 and Sycp1 expressed in gonads treated with rapamycin (Supplementary Fig. S5e). Furthermore, the number of meiocytes that underwent homologous chromosome synapsis in rapamycin-treated gonads was lower than that of the control gonads (Supplementary Fig. S5f). Moreover, similar to ISCK03 treatment, rapamycin treatment blocked the zygotene stage, with fewer cells progressing to the pachytene phase (Supplementary Fig. S5g). The rate of abnormal synapsis was greater in rapamycin-treated gonads than in control gonads (Supplementary Fig. S5h).

These findings were corroborated by western blot analysis, which revealed decreased protein levels of Stra8, Sycp1, Sycp3 and Vasa in response to rapamycin treatment (Supplementary Fig. S6a). Furthermore, the number of DSB repair-associated protein Rad51 foci was increased (Supplementary Fig. S6b), and the number of Dmc1 foci was reduced by rapamycin treatment (Supplementary Fig. S6c). The number of the crossover formation marker Mlh1 also decreased in response to rapamycin treatment (Supplementary Fig. S6d). In summary, the inhibition of mTOR delays meiotic entry and impairs homologous chromosome DSB repair and crossover.

**Fig. 6:**
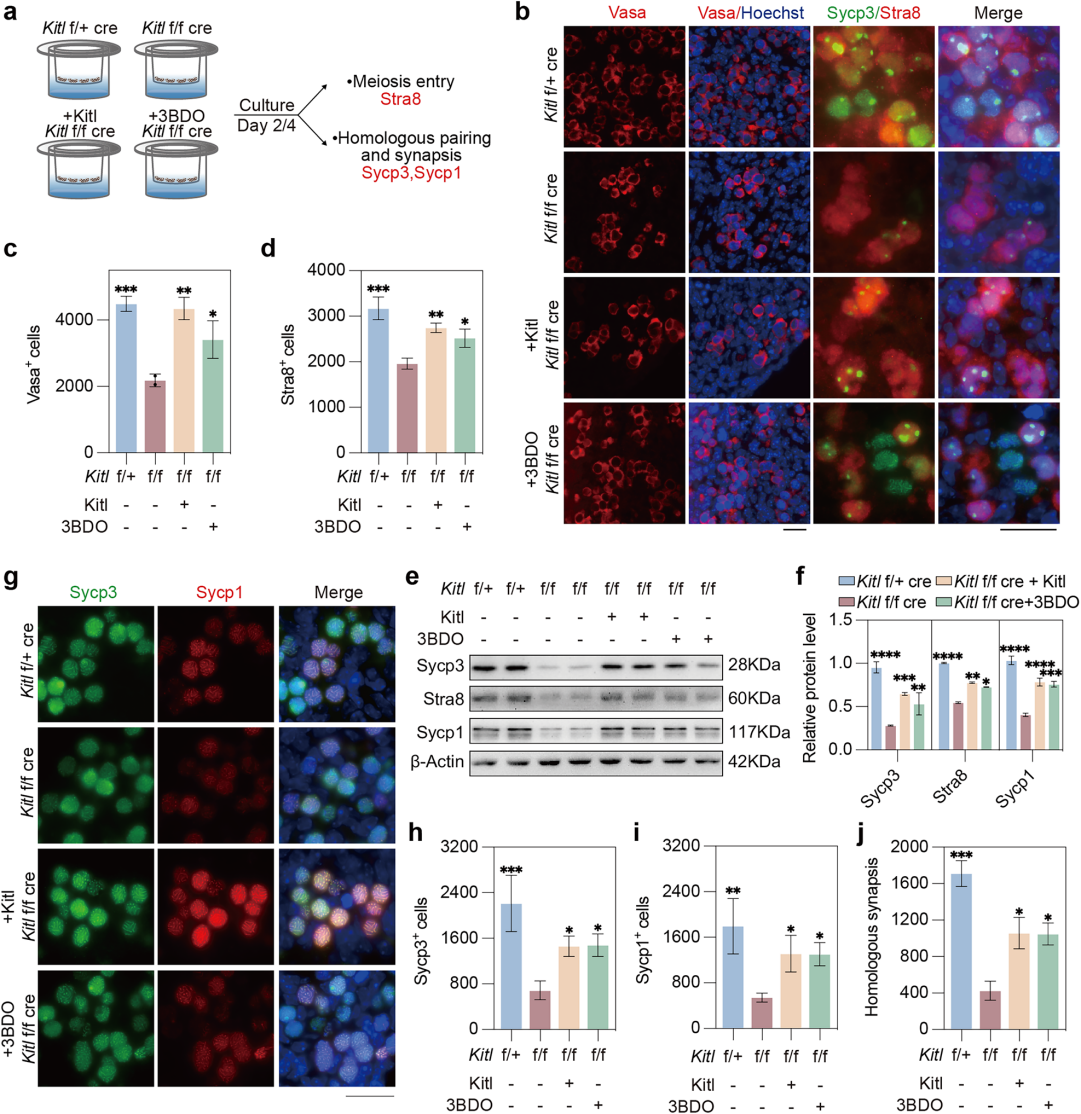
Activating mTOR alleviates meiosis defects induced by *Kitl* deficiency. **a** Experimental design involving E12.5 *Kitl* f/f cre and f/+ cre female gonads treated with or without Kitl or 3BDO as an mTOR activator for 2 or 4 days and examined for key meiosis markers. **b** Representative immunofluorescence images of Vasa (left), Stra8 and Sycp3 (right) in female gonads cultured for 2 days. Green, Sycp3; red, Vasa or Stra8; blue, nuclei counterstained with Hoechst 33342. Scale bar: 20 μm. **c** The number of Vasa^+^ cells per gonad cultured for 2 days. *n* = 3 fetal mouse gonads. **d** The number of Stra8^+^ cells per gonad cultured for 2 days. *n* = 3 gonads. Western blot (**e**) and quantification (**f**) of the Sycp3, Stra8 and Sycp1 protein levels in gonads cultured for 2 days. β-actin served as a loading control. *n* = 2 independent experiments. **g** Immunofluorescence of Sycp1 and Sycp3 in gonads cultured for 4 days. Green, Sycp3; red, Sycp1; blue, nuclei counterstained with Hoechst 33342. Scale bar: 20 μm. **h** The number of Sycp3^+^ cells per gonad cultured for 4 days. *n* = 3 gonads. **i** The number of Sycp1^+^ cells per gonad cultured for 4 days. *n* = 3 gonads. **j** Quantification of the number of cells with homologous synapsis per gonad, as evidenced by coimmunofluorescence of Sycp1 and Sycp3. *n* = 3 gonads. The data are presented as the means ± SDs. *p < 0.05; **p < 0.01; ***p < 0.001; ****p < 0.0001.

Given that inhibition of mTOR induces meiotic defects, we next asked whether the meiotic defects observed in *Kitl* cKO mice could be rescued by activating mTOR signaling. To address this, we supplemented *Kitl* cKO gonads with an mTOR activator (3BDO) or Kitl and cultured them for 2 or 4 days (Fig. 6a). The number of Vasa^+^ germ cells in the *Kitl* f/f cre gonads was lower than that in the *Kitl* f/+ cre gonads cultured for day 2. However, the addition of Kitl to the *Kitl* f/f cre gonads increased the number of germ cells, and supplementation with 3BDO resulted in a similar increase in Vasa^+^ cells (Fig. 6b, c).

To assess the effects of 3BDO on meiosis, we analyzed the expression of the meiosis initiation marker Stra8 (Fig. 6b). The abundance of Stra8^+^ germ cells was greater in the *Kitl* f/+ cre gonads than in the *Kitl* f/f cre gonads. Supplementation with Kitl partially restored meiosis initiation in *Kitl* f/f cre gonads, and the inclusion of 3BDO also alleviated the meiotic initiation defect in *Kitl* f/f cre gonads (Fig. 6d). Western blot analysis revealed that the protein levels of Stra8, Sycp1 and Sycp3 were elevated by either Kitl or the mTOR activator (Fig. 6e, f). On day 4, immunofluorescence analysis of Sycp3 and Sycp1 expression revealed that the number of Sycp3^+^ cells was significantly lower in the gonads of the *Kitl* f/f cre than in those of the *Kitl* f/+ cre, but this decrease could be partially reversed by the addition of Kitl or 3BDO (Fig. 6g, h). Similarly, the number of Sycp1^+^ cells was also reduced in *Kitl* f/f cre gonads, and supplementation with Kitl or 3BDO partially restored Sycp1 expression (Fig. 6i). Notably, while *Kitl* f/f cre exhibited a significant reduction in the number of meiocytes with normal synapsis compared with that of *Kitl* f/+ cre, this defect was partially rescued by either Kitl or 3BDO, despite not reaching the levels observed in *Kitl* f/+ cre gonads (Fig. 6j). The partial rescue effect could be attributable to the limitations of in vitro cultured gonads, which may not fully recapitulate their in vivo counterparts, and/or the potential dosage effects of Kitl or 3BDO, which can be further optimized.

Together, these results suggest that mTOR functions downstream of the Kitl/Kit signaling pathway. The Kitl-Kit-mTOR axis represents a critical mechanism in promoting meiotic initiation and progression.

### Kitl/Kit activates mTOR/p-S6 signaling by stimulating p-AKT

**Fig. 7:**
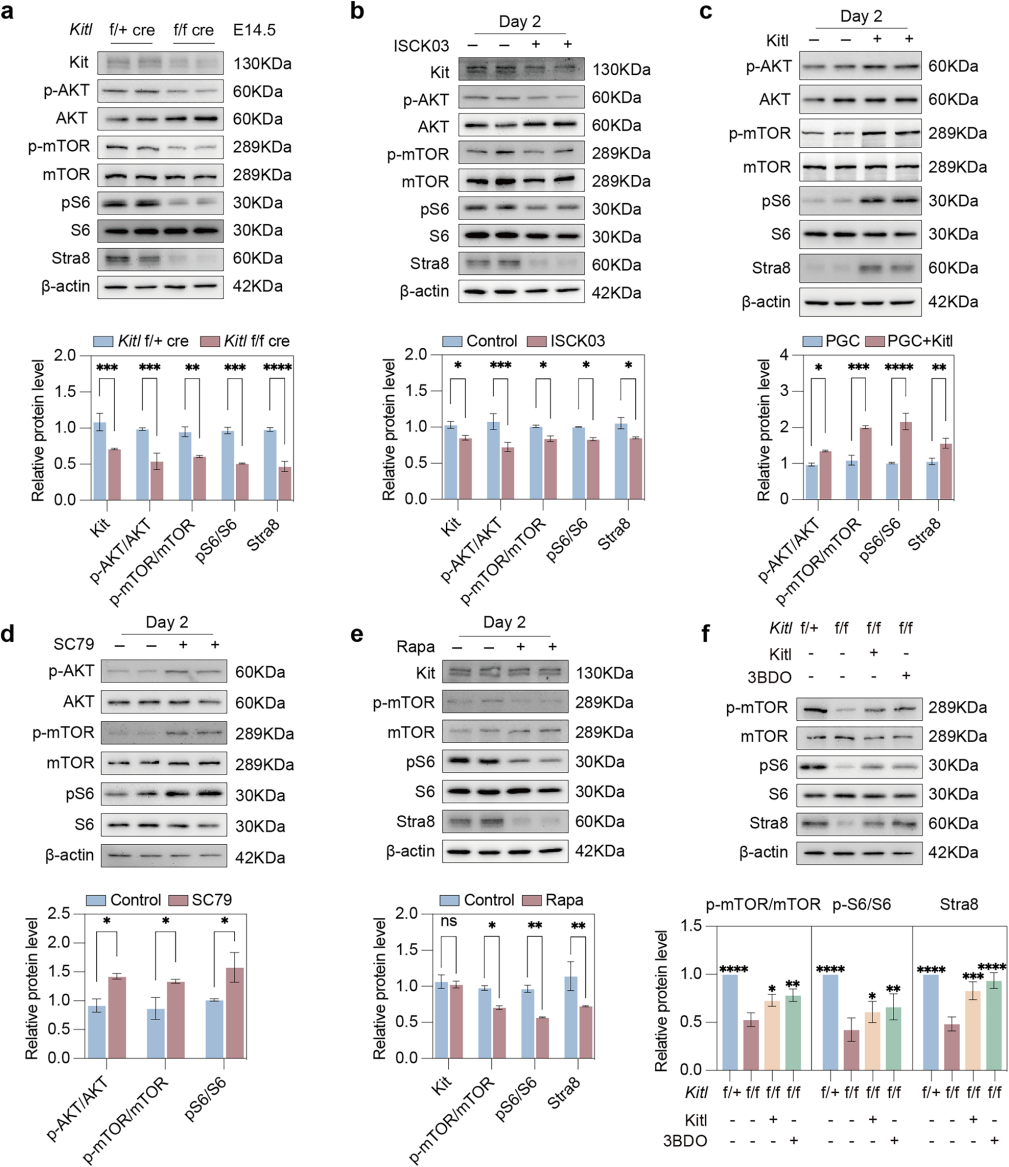
Kitl/Kit activates p-AKT to promote the mTOR signaling pathway. **a** Western blot and quantification of the Kit, p-AKT, AKT, p-mTOR, mTOR, pS6, S6, and Stra8 protein levels in E14.5 *Kitl* f/f cre and f/+ cre female gonads. **b** Western blot and quantification of the Kit, p-AKT, AKT, p-mTOR, mTOR, pS6, S6, and Stra8 protein levels in the control and ISCK03-treated (Kit inhibitor) gonads cultured for 2 days. **c** Western blot and quantification of the p-AKT, AKT, p-mTOR, mTOR, pS6, S6, and Stra8 protein levels in PGCs and Kitl-treated PGCs cultured for 2 days. **d** Western blot and quantification of the p-AKT, AKT, p-mTOR, mTOR, pS6, and S6 protein levels in the control and SC79-treated (p-AKT activator) gonads cultured for 2 days. **e** Western blot and quantification of the Kit and p-mTOR, mTOR, pS6, S6, and Stra8 protein levels in the control and Rapa-treated (Rapamycin, mTOR inhibitor) gonads cultured for 2 days. **f** Western blot and quantification of p-mTOR, mTOR, pS6, S6, and Stra8 protein levels in gonads of different groups include *Kitl* f/+ cre, *Kitl* f/f cre, 3BDO (mTOR activator) and Kitl on day 2 of culture. The data are presented as the means ± SDs. β-actin served as a loading control. *n* = 2 independent experiments. n.s., not significant, *p* > 0.05; *p < 0.05; **p < 0.01; ***p < 0.001; ****p < 0.0001.

The above data revealed that *Kitl* deficiency in granulosa cells of the fetal gonads decreased the p-AKT/AKT, Foxo3a, and p-Stat3 levels (Supplementary Fig. S4h, i). Furthermore, we validated a significant reduction in Kit protein levels, along with a marked decrease in p-AKT/AKT, p-mTOR/mTOR, and pS6/S6 levels in E14.5 *Kitl* f/f cre (cKO) gonads (Fig. 7a). Similarly, inhibition of Kit by ISCK03 decreased p-AKT/AKT, p-mTOR/mTOR, and pS6/S6 levels as well as Stra8 protein levels (Fig. 7b). In contrast, the addition of Kitl to PGCs increased p-AKT/AKT, p-mTOR/mTOR, pS6/S6, and Stra8 protein levels (Fig. 7c). Moreover, in *Kitl* f/f cre gonads treated with the p-AKT activator SC79 [55], we observed a notable increase in p-AKT/AKT levels after 2 days, accompanied by elevated p-mTOR/mTOR and pS6/S6 protein levels (Fig. 7d). These results demonstrated that Kitl/Kit activates p-AKT to promote mTOR/pS6 signaling.

Additionally, p-mTOR/mTOR, pS6/S6 and Stra8 protein levels were reduced by rapamycin treatment (Fig. 7e), whereas supplementation with Kitl or 3BDO in *Kitl* f/f cre gonads partially restored the expression levels of these proteins (Fig. 7f). Notably, while ISCK03 treatment caused a significant reduction in Kit expression (Fig. 7b), no substantial change in the Kit protein level was observed after rapamycin treatment (Fig. 7e). These data together validated the function of Kitl-Kit-AKT-mTOR-pS6 signaling in the meiosis of fetal ovaries.

### Supplemental Kitl promotes the initiation and progression of meiosis in PGCs

Meiosis entry of PGCLCs produced from pluripotent stem cells (PSCs) can be induced by RA and BMP2, but the efficiency, such as the proportion of Sycp3^+^ cells, is lower than that of fetal ovaries [18]. Given that somatic Kitl plays a pivotal role in regulating germ cell meiosis within the female gonads, we tested whether direct supplementation of Kitl could enhance the meiotic competence of PGCs in vitro.

We first analyzed RNA-seq data from Miyauchi et al. [18] to compare gene expression in cultured cells for day 9 under different treatments (RA, BMP2) with that in E14.5 female germ cells because of their transcriptional similarity. In addition to the similarity between PGCLCs and E14.5 female germ cells, we focused on their differences. PGCLCs cultured with RA exhibited increased activation of the RA, ErbB, and Hippo pathways (Supplementary Fig. S7a). In addition, PGCLCs cultured with RA and BMP2 activated the mTOR and Wnt pathways (Supplementary Fig. S7b). However, compared with E14.5 female germ cells, PGCLCs cultured with RA and BMP2 showed defects in meiosis induction, including impaired synaptic complex formation. Additionally, PGCLCs cultured with RA and BMP2 lacked activation of the Scf (Kitl)/Kit pathway (Supplementary Fig. S7c). These results indicate that RA and BMP2 induce meiosis in PGCLCs, but their efficiency is lower than that in somatic cells, with defects likely due to the absence of key pathways such as Kitl/Kit signaling.

**Fig. 8:**
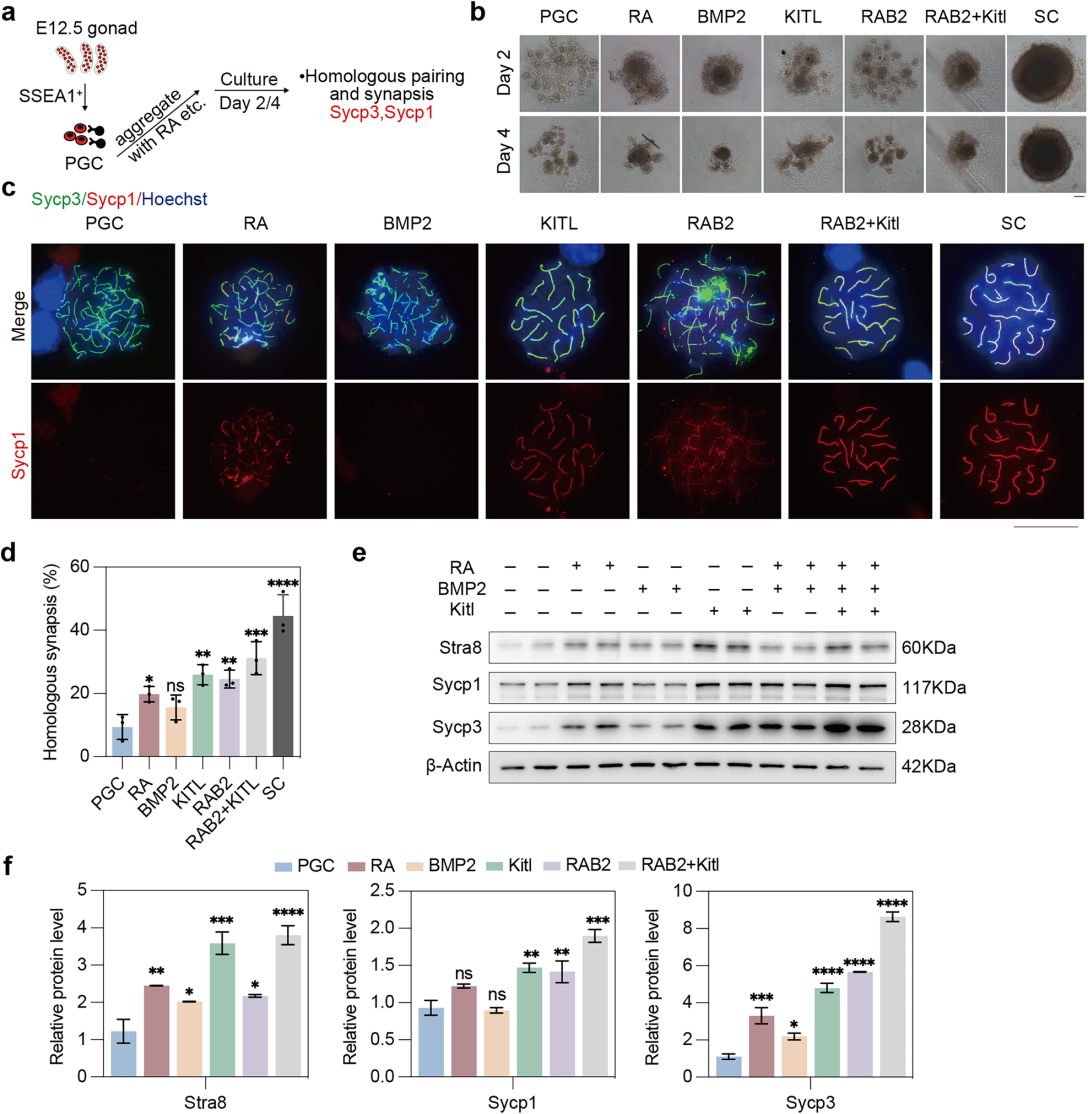
Supplementation of Kitl elevates the protein levels of Stra8, Sycp1 and Sycp3 and homologous synapsis and pairing. **a** Experimental design involving PGCs sorting from E12.5 female gonads treated with small molecules for 2 or 4 days. The groups included the PGC control, RA, BMP2, Kitl, RAB2 (RA + BMP2), RB+Kitl (RAB2+Kitl), and SC (somatic cells) groups. **b** Bright-field morphology after PGCs were sorted and subjected to different treatments. Scale bars: 100 μm. **c** Representative immunofluorescence chromosome spread images of PGCs from different groups on day 4 stained for Sycp3 and Sycp1. Green, Sycp3; red, Sycp1; blue, nuclei counterstained with Hoechst 33342. Scale bar: 20 μm. **d** Quantification of the percentage of cells with homologous synapsis, as evidenced by coimmunofluorescence of Sycp1 and Sycp3, in different groups of PGCs cultured for 4 days. *n* = 3 independent experiments. Western blot (**e**) and quantification (**f**) of the Stra8, Sycp1 and Sycp3 protein levels in PGCs from different groups on day 2. β-actin was used as a loading control. *n* = 2 independent experiments. The data are presented as the means ± SDs. n.s., not significant, *p* > 0.05; *p < 0.05; **p < 0.01; ***p < 0.001; ****p < 0.0001.

Hence, we conducted experiments to compare the effects of RA, BMP2, Kitl, RAB2 (RA + BMP2), RAB2+Kitl, and somatic cells (SC) on the entry of PGCs into meiosis. PGCs were isolated from E12.5 female gonads via SSEA1 magnetic beads and cultured under these conditions (Fig. 8a). Meiotic progression was assessed on the second and fourth days. Upon the addition of RAB2 and Kitl, the PGCs aggregated into more compact structures resembling somatic cells than did those without RAB2 or Kitl (Fig. 8b).

Moreover, by immunofluorescence, unlike with somatic cells, RA only partially activated Sycp1 protein expression, RAB2, Kitl, and RAB2 + Kitl increased the protein expression of Sycp1, whereas BMP2 alone did not induce Sycp1 protein expression (Fig. 8c). On day 4, the addition of Kitl promoted normal synapsis in many meiotic cells compared with that in untreated PGCs, which served as controls. RA also facilitated synapsis but less efficiently than did Kitl. Additionally, the combination of RAB2 + Kitl further enhanced meiotic progression, suggesting that RA and BMP2 are insufficient for meiosis initiation and that meiosis progression requires further activation by Kitl (Fig. 8d). Western blotting analysis corroborated the findings revealed by immunofluorescence (Fig. 8e). Both RA and BMP2 can induce the expression of Stra8 and Sycp3, either alone or in combination. However, RA or BMP2 alone cannot increase Sycp1 protein levels. Elevated Stra8 and Sycp1 protein expression was observed in the presence of RAB2 and/or Kitl. These data further demonstrate that Kitl can effectively initiate meiotic entry and promote the expression levels of key meiotic proteins in vitro (Fig. 8f).

To further investigate potential crosstalk between these pathways, we examined RA pathway-related genes reported by published work [11, 14], including *Aldh1a1*, *Aldh1a2*, *Aldh1a3*, *Rara*, *Rarb*, and *Rarg* in *Kitl* cKO models, compared with WT by qPCR and found no significant expression changes (Supplementary Fig. S7d). Similarly, neither mTOR inhibition (rapamycin treatment) nor activation (3BDO/Kitl supplementation) altered RA pathway gene expression (Supplementary Fig. S7e, f). These results suggest that the Kitl signaling acts on the meiotic entry largely independent of RA, despite that the potential feedback regulation cannot be excluded.

## Discussion

We report the crucial role of the Kitl/Kit signaling pathway in somatic cell–germ cell interactions for meiotic entry and progression in the fetal mouse ovary. Moreover, Kitl/Kit promotes mTOR/p-S6 signaling by activating the p-AKT pathway. Consistently, p-AKT has been shown to activate mTOR in other cell types [56]. Furthermore, our study revealed important functions of the mTOR pathway in promoting sufficient protein levels that are key for meiosis initiation, homologous synapsis and recombination, including Stra8, Sycp1, Sycp3 and Vasa. The Stra8 protein is known to promote the transcription of key meiosis genes for homologous synapsis, pairing, and recombination [22, 57, 58]. Elevated levels of the Stra8 protein promoted by Kitl/Kit–pAKT–mTOR/pS6 also explain why the transcription of meiosis genes downstream of Stra8 and related signaling pathways is downregulated in *Kitl* cKO gonads.

Previously, Kitl/Kit was shown to function in various cell types, including hematopoietic stem cells, mast cells, melanocytes, and cancers [59–61]. During germ cell development, Kitl/Kit is required for oocyte growth and survival in vitro [62, 63], follicular activation and development [44], PGC migration and survival during embryogenesis [64, 65], and spermatogonial proliferation and differentiation [66]. Kitl/Kit also regulates FoxO3 to control primordial follicle activation [67]. Kit expression in PGCs has been shown to promote the survival and proliferation of PGCs in the fetal ovary [68–71]. However, Kitl (SCF) may not promote meiosis entry (PGC differentiation) on the basis of the reduced expression of Kit after PGCs enter meiosis [72]. These previous studies were mostly based on immunofluorescence staining, which may not reflect the true function of the Kitl/Kit signature. In vitro studies have shown that the addition of Kitl to spermatogonial culture systems can promote entry into meiosis [73]. Yet, it is suggested that signaling via the Kitl/Kit prevents meiotic entry and progression within the gonads [74]. Hence, the specific roles and mechanisms of Kitl/Kit signaling in meiosis entry and homologous synapsis, pairing, and recombination remain elusive. Here, we show important roles of Kitl in the entry and progression of meiosis in the mouse fetal ovaries, by taking advantage of various advanced comprehensive approaches, including single-cell RNA-seq, conditional knockout, and extensive western blot analysis. Moreover, we uncover a previously unknown mechanism by which mTOR signaling is involved in Kit-induced meiosis entry and progression by promoting the expression of key meiosis proteins, including Stra8, Sycp1, and Sycp3.

In summary, our study reveals critical roles for the Kitl/Kit/AKT/mTOR/pS6 signaling axis in promoting sufficient protein levels of critical genes for meiotic initiation and progression (Supplementary Fig. S8). This pathway operates in parallel with the RA-dependent transcriptional activation of *Stra8*, providing a robust and multilayered regulatory system to ensure the proper timing and execution of meiosis. These findings highlight the critical role of somatic cell‒germ cell interactions in meiotic regulation and may open new avenues for understanding the molecular mechanisms underlying meiosis initiation and progression in female meiosis.

While our study highlights the role of Kitl in regulating meiosis through the mTOR/pS6 pathway and its effects on protein levels of key meiosis genes, including Stra8, Sycp1, Sycp3 and Vasa, and on mRNA expression, several limitations remain. Additional mechanisms linking Kitl/Kit signaling to AKT/mTOR/pS6 activation and the potential involvement of intermediate factors warrant further investigation. Additionally, although we observed changes in *Stra8* mRNA and other meiosis-related genes, such as *Spo11* and *Meioc*, the temporal regulation of these events at both the transcriptional and translational levels requires further experimentation. Other signals from somatic cells that may interact with the Kitl/Kit and AKT/mTOR/pS6 pathways during meiotic initiation and progression cannot be excluded.

## Materials and methods

### Mice

The use of mice for this research was approved by the Nankai University Animal Care and Use Committee. All the mice were maintained in the specific pathogen-free animal facility of the Experimental Animal Center, Nankai University, with a humidity of 35± 4%, a stable temperature of 24 ± 1 °C, and a 12/12 h light/dark cycle. Six-week-old female mice were subsequently mated with 8-week-old male mice (2:1) overnight, and the vaginal plug was checked the next morning. The mice with a vaginal plug were considered 0.5 days postcoitum (dpc). *Kitl*^flox/flox^; *Foxl2*-Cre female mice were obtained by crossing *Kitl*^flox/flox^; *Foxl2*-Cre male mice with *Kitl*^flox/+^; *Foxl2*-Cre female mice in a C57BL/6 background. At least 3–4 mice per group were used for each experiment. All animals were humanely euthanized by cervical dislocation for tissue collection. Death was further assessed by confirmation of rigor mortis according to AVMA Guidelines for the Euthanasia of Animals. All efforts were made to minimize the number of animals used and their suffering.

### Genotyping

PCR genotyping was performed on the extracted DNA. The sequences of the primers used are also shown in Table S4.

*Foxl2*-Cre-Forward, 5′-CGGCATGGTGCAAGTTGAAT-3′

*Foxl2*-Cre-Reverse, 5′-TCAGCTACACCAGAGACGGA-3′

*Kitl*-flox-Forward, 5′-CGAGGTAGGGGAAAAGAACC-3′

*Kitl*-flox-Reverse, 5′-GGATCTTCCCAGAGGTTGGA-3′

### Sample collection

*F*_1_ embryos were harvested daily at E12.5–E16.5 from the date of the vaginal plug, with further morphological verification at dissection. The embryonic urogenital complexes were carefully removed under a stereomicroscope and washed in cold PBS twice. The embryonic gonads were separated from adjacent mesonephros for further culture with different small molecules or fixed with 4% paraformaldehyde (PFA) at 4 °C to obtain frozen sections.

### Magnetic‑activated cell sorting (MACS)

Magnetic activated cell sorting (MACS) was performed according to the manufacturer’s instructions (Miltenyi). Briefly, the female gonads of E12.5 were digested with trypsin at 37 °C for 7 min, after which the digestion was terminated with 1 mL of culture mixture containing serum, washed once with MACS buffer, and finally, the samples were suspended in 80 µL of MACS buffer and incubated with 20 µL of SSEA1 magnetic beads at 4 °C for 20 min. After the mixture was cleaned with MACS buffer, 1 mL was resuspended and filtered through an MS column. The somatic cells were directly discharged, and the germ cells were bound to the magnetic beads. The sorted gonadal somatic cells and MACS-sorted PGCs were plated in the wells of a low-cell-binding U-bottom 96-well Lipidure-Coat plate in MF10 medium (MF10 medium containing M199 (Sigma) supplemented with 10% FBS, 1 mM L-glutamine, 1% nonessential amino acid stock, 50 units/mL penicillin and 50 mg/mL streptomycin, 50 mg/mL VC, and 10 mM Rocki).

### In vitro gonad culture

Embryonic gonads collected from embryos were placed on Transwell membranes (Millipore) and cultured in MF10 medium (as described above) for 2 or 4 days. For specific experiments, small molecules (ISCK03, rapamycin, 3BDO, SC79, and Kitl) were added to MF10 medium at the concentrations detailed in Table S3.

### Immunofluorescence microscopy of female gonads

After the gonads were removed, they were washed once with PBS, fixed at 4 °C overnight with 4% PFA, dehydrated with 30% sucrose for 2 h, and then embedded in optimal cutting temperature (OCT) ice. Frozen sections were cut at a thickness of 5 μm. After the slides were left at room temperature for 30 min, they were washed in PBS for 10 min, fixed with precooled acetone for 15 min, dried in a fume hood for 10 min, and permeabilized for 30 min in PBS containing 0.1% Triton X-100. After the slides were blocked with 3% BSA for more than two hours, the corresponding primary antibodies (Vasa, ab13840, Abcam, 1:200; Vasa, ab27591, Abcam, 1:200; Sycp1, ab15090, Abcam, 1:200; Sycp3, ab97672, Abcam, 1:200; Sycp3, NB300--230, Novus, 1:200; Stra8, ab49602, Abcam, 1:200; Kit, AF1356, R&D, 1:200; Kitl, ab64677, Abcam, 1:200; Foxl2, ab5096, Abcam, 1:200; Cleaved caspase-3, 9961, CST, 1:200) were incubated with the samples at 4 °C overnight. On the second day, the slides were washed with PBS for 15 min three times and then incubated with the appropriate fluorescence-conjugated secondary antibodies (donkey anti-goat IgG (H + L), Alexa Fluor 594, A-11058, Invitrogen, 1:200; donkey anti-mouse IgG (H + L), Alexa Fluor 488, A-21202, Invitrogen, 1:200; donkey anti-rabbit IgG (H + L), Alexa Fluor 594, A-21207, Invitrogen, 1:200; donkey anti-mouse IgG (H + L), Alexa Fluor 594, A-21203, Invitrogen, 1:200) at room temperature in the dark for 1.5 h. After the slides were washed with PBS for 15 min three times, they were counterstained with 0.5 mg/mL Hoechst 33342 in Vectashield (VectorLabs) mounting medium. Fluorescence was detected and imaged via a Carl Zeiss Axio-Imager Z2 fluorescence microscope or Zeiss LSM710 laser scanning confocal microscope. The specific antibodies used are listed in Table S2.

### Immunofluorescence microscopy of meiocyte spreads

The surface spreads of meiocytes were prepared via a drying-down technique and stained for synaptonemal complexes [46]. In brief, the gonads or aggregates were collected at different times and digested at 37 °C with 0.25% TE for 7 min, after which 100 mM sucrose was added, and the mixture was evenly and slowly added to the slides with 1% PFA. The mixture was allowed to dry at room temperature for 3 h and then washed with 1% photoflow (Kodak) for 1 min. The dried slides were washed with PBS containing 0.1% Triton X-100 for 10 min. Then, immunofluorescence was performed as described above with the corresponding primary antibodies (Sycp3, ab97672, Abcam, 1:200; Sycp1, ab15090, Abcam, 1:200; Sycp3, NB300-230, Novus, 1:200; Rad51, ab133534, Abcam, 1:300; Dmc1, ab11054, Abcam, 1:200; Mlh1, Proteintech, 11697-1-AP, 1:100). Fluorescence was detected and imaged via a Carl Zeiss Axio-Imager Z2 fluorescence microscope, a confocal laser scanning microscope (LSM710) or STEDYCON (Abberior Instruments). For each genotype or experimental group, at least 30 fields of view were analyzed, and the experiment was repeated at least three times. The final data represent the percentage of cells at specific meiotic stages. The specific antibodies used are listed in Table S2.

### Gonad fixation and processing

Gonads were collected from embryos at E12.5--E16.5 or from in vitro culture experiments. For embryonic gonads, tissues were dissected at the specified time points, washed twice with PBS, and immediately fixed in 4% PFA at 4 °C overnight. For cultured gonads, tissues were carefully removed from Transwell membranes to preserve morphology, washed twice with PBS, and fixed in 4% PFA at 4 °C overnight. The following day, the 4% PFA was replaced with 30% sucrose for complete dehydration. Once the gonads sank to the bottom of the tube, they were embedded in OCT compound, frozen in liquid nitrogen, and stored at −80 °C until sectioning. Sections were cut at a thickness of 5 µm. Starting from the first section, every fifth section was collected until the entire gonad was sectioned.

For immunofluorescence, sections were stained with target antibodies, and positive cells were counted across the entire gonad. The final data represent the total number of positive cells per gonad.

### TUNEL in combination with immunofluorescence staining

Apoptotic cells in female mouse gonads were detected using the Vazyme TUNEL BrightRed Apoptosis Detection Kit (A113, Vazyme Biotech) according to the manufacturer’s instructions with minor modifications. Briefly, gonad sections were treated with PBST (0.2% Triton X-100 in PBS) for 20 min at room temperature. Following three washes with PBS, sections were incubated with the TUNEL reaction mixture for 1 h at 37 °C in a humidified chamber protected from light. As a positive control, selected sections were pretreated with DNase I (20 U/mL) for 10 min at room temperature prior to the TUNEL reaction to induce artificial DNA strand breaks. After TUNEL labeling, sections were further processed for germ cell marker Vasa (Ddx4) immunofluorescence as described in the immunofluorescence method, to assess apoptosis in germ cells.

### Western blot

The tissues were digested with 0.25% TE, washed once in PBS, lysed on ice with lysis buffer for 30 min, and then sonicated for 2 min every 2 s at an amplitude of 60. After centrifugation at 10,000×*g* at 4 °C for 10 min, the supernatant was transferred to a new tube. The concentration of protein samples was determined via BCA, and the protein samples were boiled with SDS sample buffer at 95 °C for 10 min. Next, 3 μg of protein was electrophoresed via 8% or 10% SDS‒PAGE and transferred to polyvinylidene fluoride (PVDF) membranes (Millipore) via the Mini Trans-Blot system (Bio‒Rad). Nonspecific binding was blocked in 5% skim milk in TBST at room temperature for 2 h or 4 °C overnight. The blots were then probed with primary antibodies (Vasa, ab13840, Abcam, 1:1000; Sycp1, ab15090, Abcam, 1:1000; Sycp3, ab97672, Abcam, 1:1000; Stra8, ab49602, Abcam, 1:1000; Kitl, ab64677, Abcam, 1:1000; Kit, AF1356, R&D, 1:1000; p-mTOR, 5536 T, CST, 1:1000; mTOR, 2983T, CST, 1:1000; p-AKT, 2965S, CST, 1:1000; AKT, 4691S, CST, 1:1000; p-Stat3, ab76315, Abcam, 1:1000; Foxo3a, 12829, CST, 1:1000; p-ERK, 9101S, CST, 1:1000; and ERK, SC93, Santa Cruz, 1:2000), and β-actin (P30002, Abmart, 1:50,000) was used as a loading control. The immunoreactivity bands were then detected, and the samples were incubated at room temperature for 2 h with the appropriate secondary antibodies. The protein bands were detected with a chemiluminescent HRP substrate. The specific antibodies used are listed in Table S2. Original blot pictures are shown in the Supplementary Material.

### RNA extraction and quantitative real‑time PCR (qPCR)

Total RNA was extracted with an RNA mini kit, and RNA was reverse transcribed into cDNA with oligo (dT) and M-MLV reverse transcriptase (Invitrogen). Real-time quantitative PCR was subsequently performed with Fast Start Universal SYBR Green Master Mix (Roche) on a real-time PCR detection system (Bio-Rad). *Gapdh* was used as an internal control, and 2^−ΔΔCt^ was used as a statistical method to detect target genes. The primer sequences are listed in Table S4.

### RNA-seq data processing

The GSE94136 dataset from Miyauchi et al. [18] was downloaded for analysis. With an adjusted *p* value < 0.05, genes with |log_2_(fold change)| > 1 were defined as significantly differentially expressed genes. GO and KEGG analyses were performed via Metascape.com, and only enriched pathways that presented a *p*-value < 0.05 were considered significantly enriched.

### Single-cell RNA sequencing computational analysis

Single-cell RNA sequencing data were processed via the Cell Ranger pipeline (10x Genomics, version 7.1.0). Raw FASTQ files were aligned to the mouse mm10 reference genome (Ensembl) via the cellranger “count --id=sample --transcriptome=mm10 --fastqs=path”. The raw count matrix is imported into R for further processing. Run R scripts via R Studio for hierarchical clustering and PCA. The count matrix was initially normalized by the library size and logarithmically transformed by Seurat 5.1.0. [42] The transcriptomes of <1000 expressed genes were discarded, while cells with mitochondrial genes occupying > 25% of the reads were defined as low-quality cells and filtered out. Seurat’s “Merge” function is used to integrate different samples according to the instructions. Unified manifold approximation (U-MAP) is used for visualization and clustering. The “FindAllMarkers” function was used to identify specific cell type markers under different conditions. The “FindMarkers” function was used to identify DEGs in germ cells between different samples, and the “VlnPlot” function was used to map the expression of target genes. Cell‒cell communication networks were inferred via the CellChat R package (version 1.6.1). The normalized gene expression matrix and cell type annotations were used as inputs. The analysis was performed sequentially via the following key functions with default parameters: createCellChat to initialize the CellChat object, subsetCommunication to filter interactions, computeCommunProb to calculate communication probabilities, computeCommunProbPathway infer signaling pathways, and netAnalysis_signalingRole and netVisual_aggregate to visualize communication networks and patterns [39]. Genes with a multiple of |log_2_(fold change)| > 0.58 and a *p* value < 0.05 were considered DEGs. The genes differentially expressed between *Kitl* f/f cre germ cells and *Kitl* f/+ cre germ cells are listed in Table S1.

### Gene set enrichment analysis (GSEA)

For single-cell RNA sequencing (scRNA-seq) data, the raw gene expression matrix was preprocessed via Seurat (v5.1.0), including normalization and log-transformation. The processed expression matrix was then used as the input for GSEA. For the bulk RNA sequencing data, GSEA was performed directly on the downloaded gene expression matrix. GSEA was performed via GSEA software (version 4.3.3) to identify enriched gene sets in the ranked gene list. The analysis was conducted via the Molecular Signatures Database (MSigDB) gene sets with default parameters, including 1000 permutations for significance testing. The enrichment results were considered significant at a *p*-value < 0.05.

### Quantification and statistical analysis

For two-group comparisons, normality within each group was assessed using the Shapiro-Wilk test alongside variance homogeneity evaluation via Levene’s test. Standard Student’s *t*-tests were applied when both groups met normality and homoscedasticity assumptions, while Welch’s *t*-tests were used for normally distributed data with unequal variances. The Wilcoxon signed-rank test was employed when either group violated normality. For multi-group comparisons, distributional properties were first evaluated through Shapiro–Wilk normality testing and Levene’s variance homogeneity assessment. One-way ANOVA was performed when all groups satisfied both normality and homoscedasticity criteria; otherwise, the Kruskal–Wallis nonparametric test was utilized. This complete analytical pipeline was implemented through custom computational scripts. Statistically significant values of *p* < 0.05, *p* < 0.01, *p* < 0.001 and *p* < 0.0001 are indicated by asterisks (*), (**), (***), and (****), respectively. The statistical methods corresponding to each figure panel are reported in Table S5.

Investigators were not blinded to allocation during the experiments, use of animals or outcome assessment. For the phenotype experiment, animals were assigned to experimental groups based on their genotype, confirmed via PCR of tail DNA, not by randomization. All other conditions (age, housing) were kept consistent across groups to minimize confounding variables. For the in vitro culture experiment using wild-type embryonic gonads, samples were randomly distributed between experimental groups, and no statistical method was used to predetermine sample size.

### Reporting summary

Further information on research design is available in the Nature Research Reporting Summary linked to this article.

## Supplementary information

reporting summary

Supplementary information

Supplementary Table S1

Original western blots

## Data Availability

The data reported in this paper are available at the NCBI Gene Expression Omnibus (GEO) with the accession numbers GSE181501 and GSE283280. GSE94136 from Miyauchi H. [18], GSE128553 from Ge W. [40], and GSE136441 from Niu W. [41] were downloaded for analysis. Any additional information required to reanalyze the data reported in this paper is available upon request to the corresponding authors.
