# Supplementary material for: Somatic Kitl promotes mTOR to facilitate prophase I of meiosis in female embryonic gonads
Source: Cell Death Dis. 2025 Nov 17;16(1):838. doi: 10.1038/s41419-025-08158-y (PMC12623929; doi:10.1038/s41419-025-08158-y)
Supplement: Supplementary file 2 — Supplementary information [file 41419_2025_8158_MOESM2_ESM.docx]

**Somatic Kitl promotes mTOR to facilitate prophase I of meiosis in female embryonic gonads**

Supplementary Information

Supplementary Figures S1–8

Supplementary Table S1 is provided separately. Genes differentially expressed between *Kitl* f/f cre and f/+ cre germ cells.

Supplementary Table S2–5


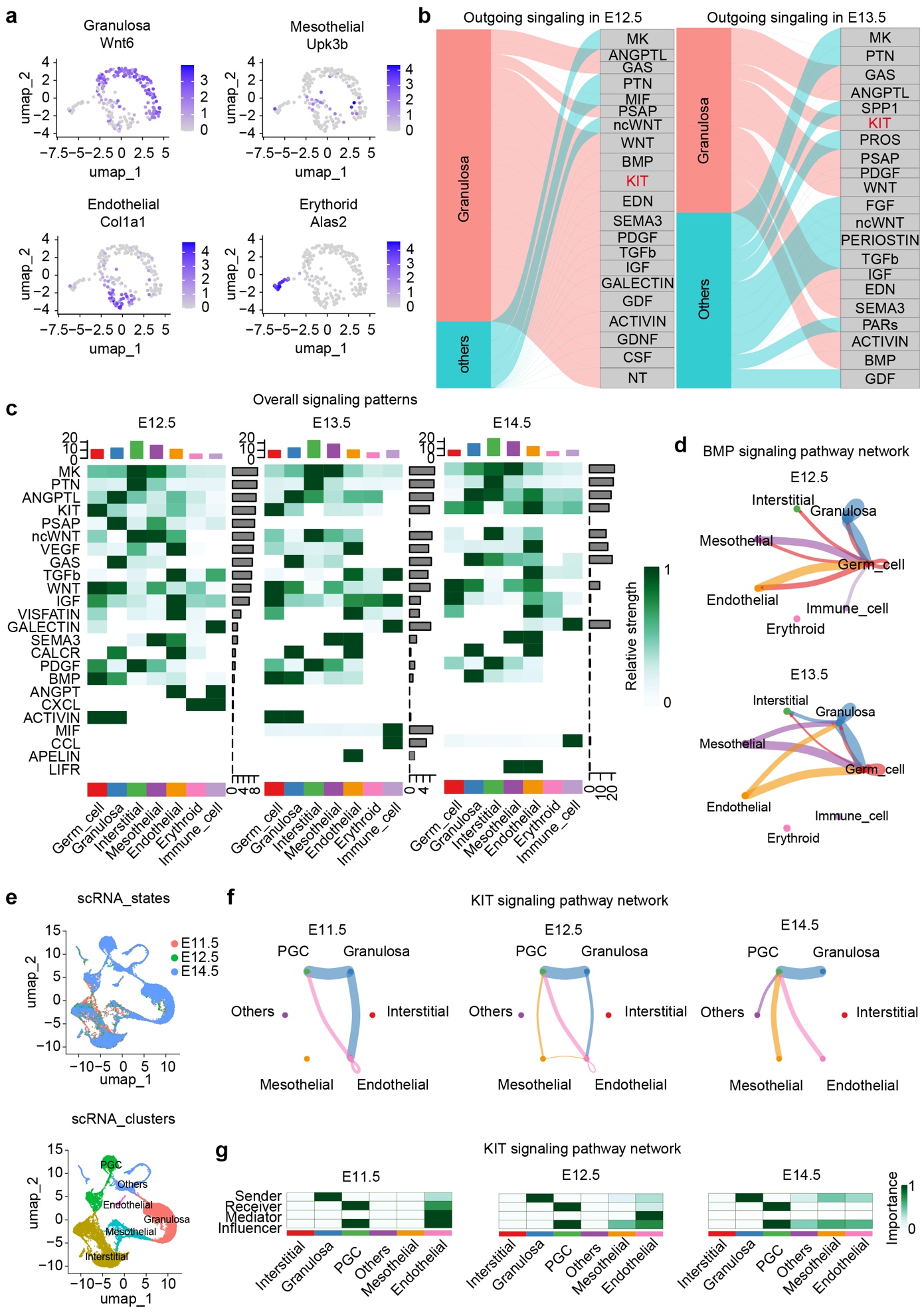


**Fig. S1 The Kitl/Kit signaling pathway is enriched between germ cells and somatic cells.**

**a** FeaturePlots of the expression levels of Granulosa (*Wnt6*), Mesothelial (*Upk3b*), Endothelial (*Col1a1*) and Erythroid (*Alas2*) cell markers from dataset A: GSE181501 in embryonic (E12.5 and E13.5) gonads.

**b** River plot showing the overall outgoing signaling pathway in E12.5 and E13.5 female gonads from dataset A.

**c** Heatmap showing the overall interaction signaling pathway in E12.5, E13.5 and E14.5 female gonads from dataset B: GSE128553 [40]. Dark green represents strong interactions, and light green indicates weak interactions.

**d** Interaction of the BMP signaling pathways in E12.5 and E13.5 female gonads, based on dataset B [40].

**e** 2D visualization of clusters based on different samples (top) and transcriptional patterns (bottom) via UMAP in E11.5, E12.5 and E14.5 female gonads, based on dataset C: GSE136441 [41].

**f** KIT signaling pathway network in E11.5, E12.5 and E14.5 female gonads from dataset C [41].

**g** Heatmap shows the role (Sender secretes ligands, Receiver expresses receptor, Mediator modulates signal transduction, and influencer orchestrates global communication) of each cell group in KIT signaling network, based on dataset C [41].


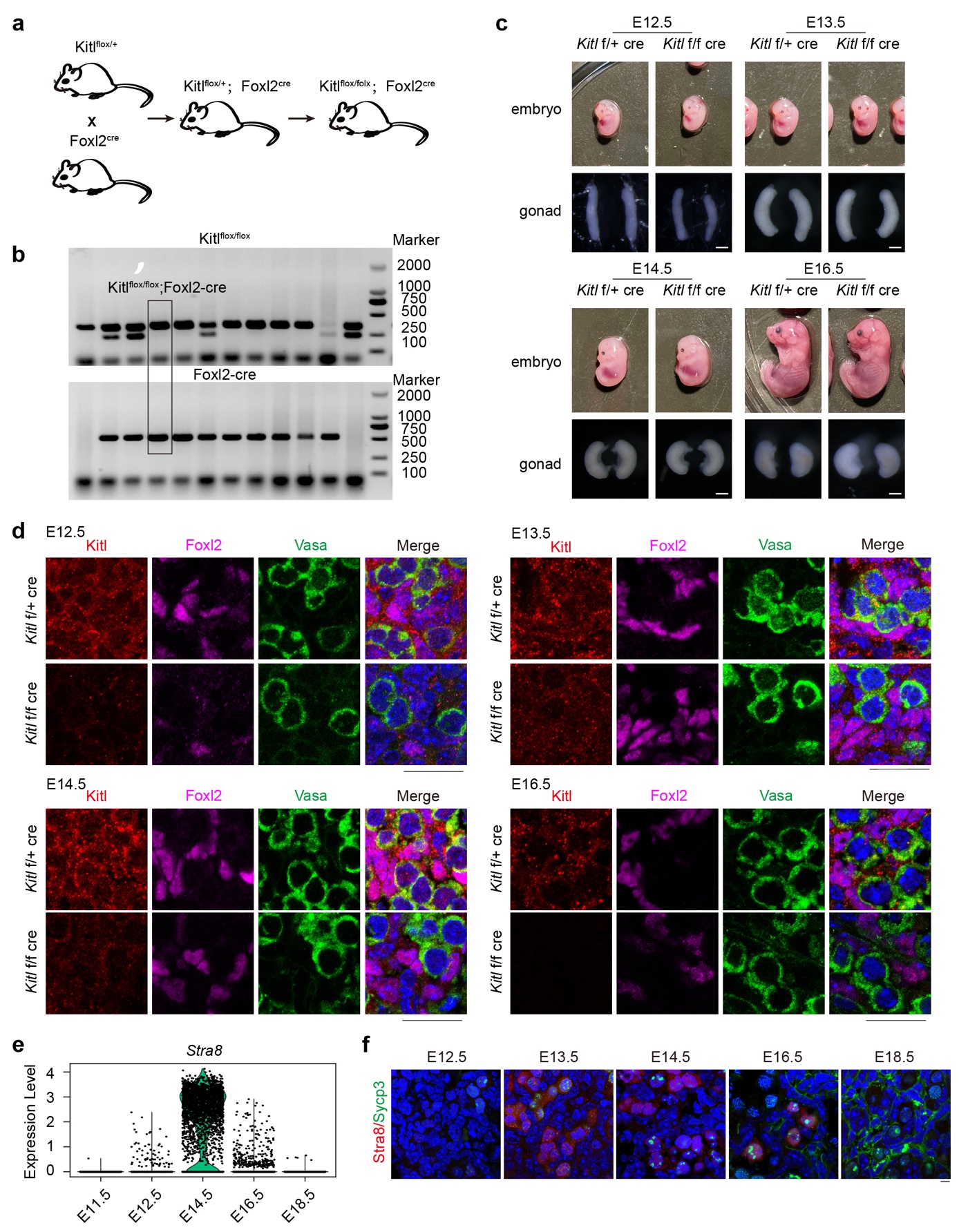


**Fig. S2 Generation of granulosa cell–*Kitl* conditional knockout (cKO) mice.**

**a** Schematic illustration showing the procedures for the generation of *Kitl* conditional knockout mice.

**b** Representative PCR genotyping analysis of *Kitl*^flox/flox^; *Foxl2*-Cre mice is demarcated by black borders.

**c** Morphology of embryos and female gonads from *Kitl* f/f cre and f/+ cre mice at E12.5, E13.5, E14.5 and E16.5. Scale bar: 50 μm.

**d** Immunofluorescence of Kitl, Foxl2 and Vasa in E12.5, E13.5, E14.5 and E16.5 *Kitl* f/f cre and f/+ cre female mouse gonads. Magenta, Foxl2; red, Kitl; green, Vasa; blue, nuclei counterstained with Hoechst 33342. Scale bar: 20 μm.

**e** Violin plot showing *Stra8* RNA levels via scRNA-seq of female gonads at various embryonic time points (E11.5--E18.5) from dataset C [41].

**f** Immunofluorescence images of Stra8 and Sycp3 in E12.5, E13.5, E14.5, E16.5, and E18.5 embryonic female gonads. Red, Stra8; green, Sycp3; blue, nuclei. Scale bar: 5 μm.


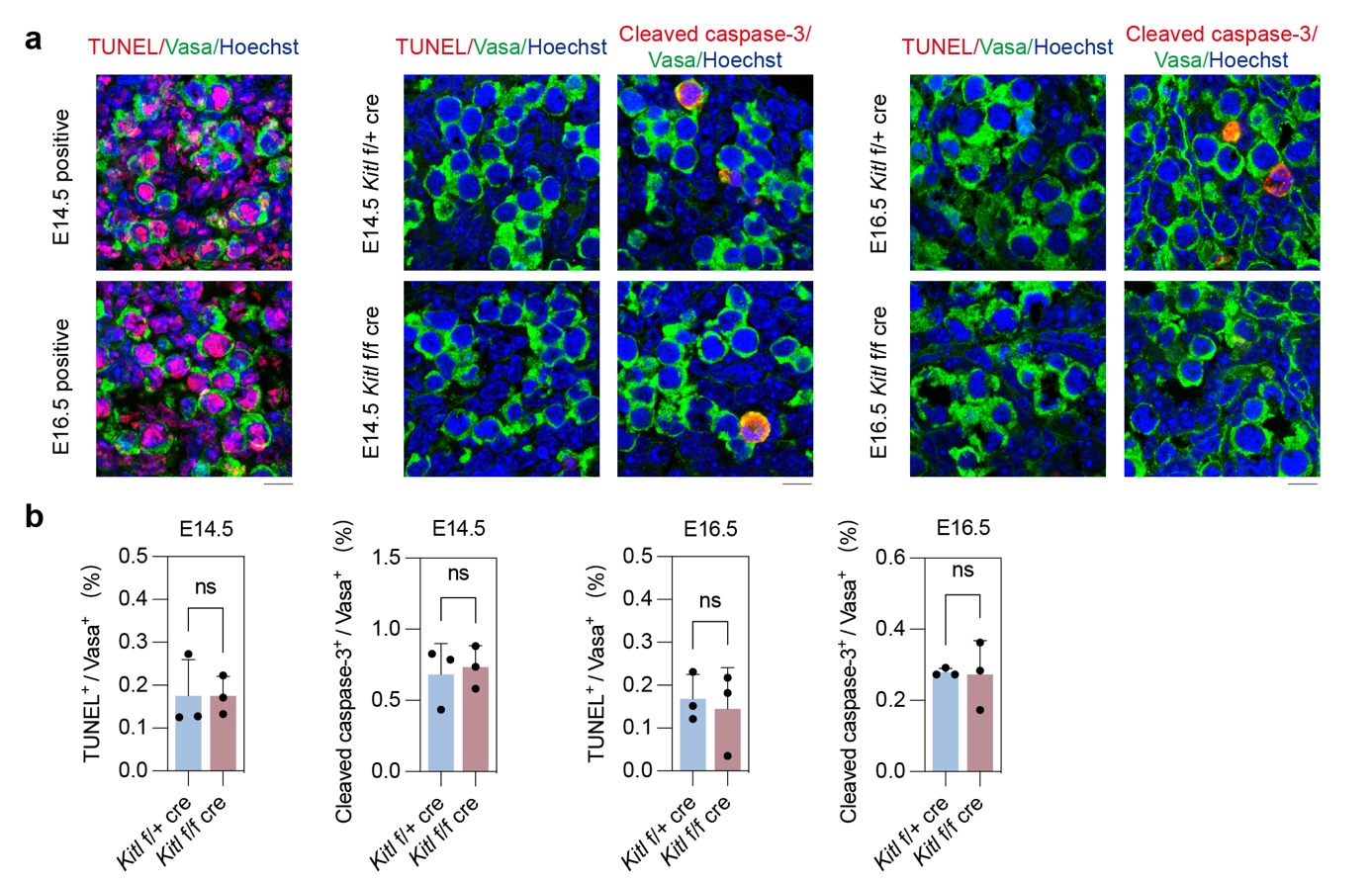


**Fig. S3 *Kitl* deficiency does not increase germ cell apoptosis**

**a** Representative immunofluorescence images of TUNEL (left), Vasa and Cleaved caspase-3 (right) in E14.5/E16.5 *Kitl* f/+ cre and *Kitl* f/f cre female gonads (Positive control: DNase I-treated samples). Green, Vasa; red, TUNEL or Cleaved caspase-3; blue, nuclei counterstained with Hoechst 33342. Scale bar: 10 μm. As a positive control, selected sections were pretreated with DNase I (20 U/mL) prior to the TUNEL reaction to induce artificial DNA strand breaks.

**b** Quantification of apoptotic (TUNEL^+^/ Vasa^+^ % or Cleaved caspase-3^+^ / Vasa^+^ %) germ cells per gonad. The data are presented as the means ± SDs. n.s., not significant.


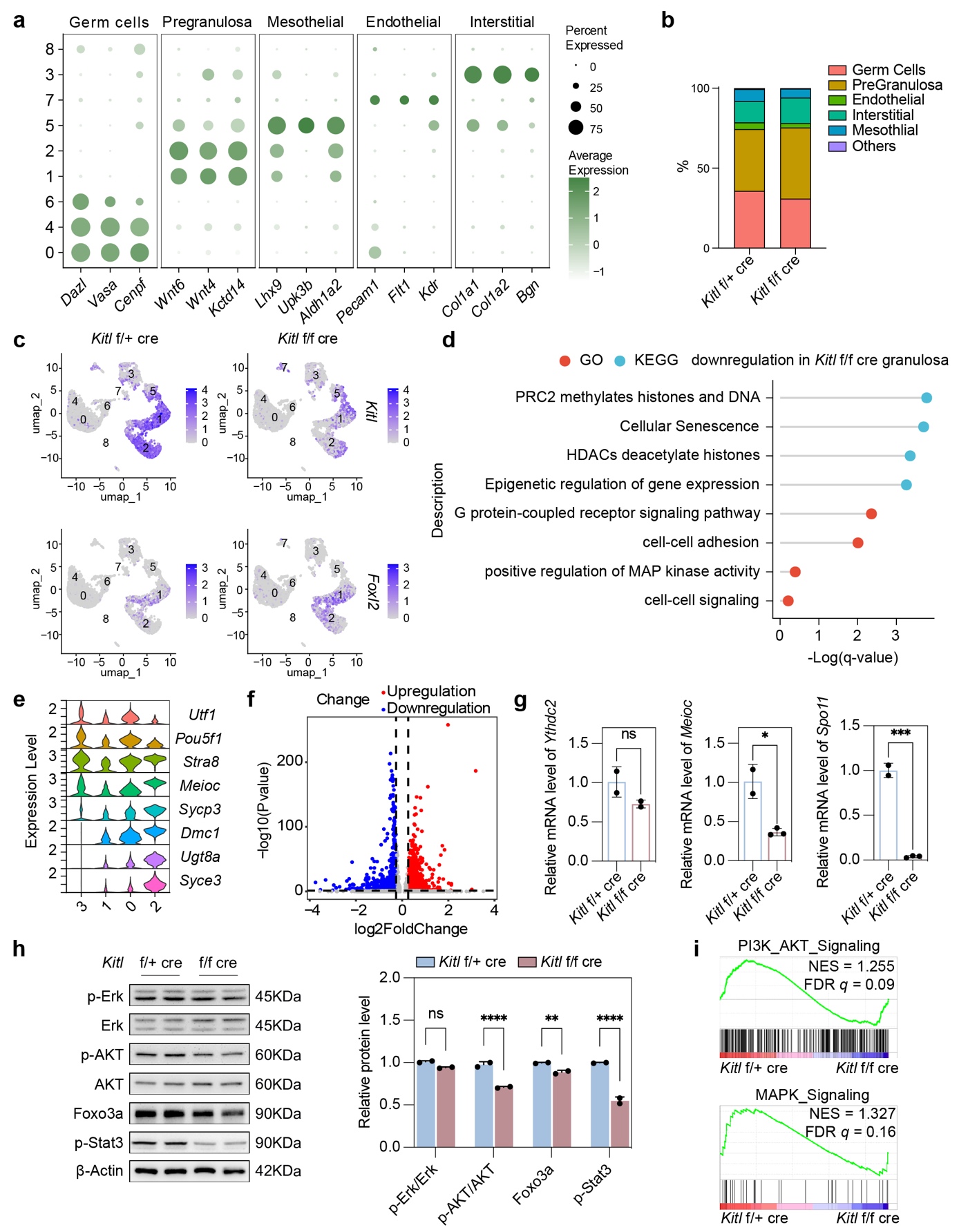


**Fig. S4 Single-cell transcriptome analysis of fetal gonads with *Kitl*-deficient granulosa cells.**

**a** Dot plot of germ cell, pregranulosa cell, mesothelial cell, endothelial cell and interstitial cell marker expression across all cell types. The dot size represents the percentage of cells expressing the indicated genes in each cluster, and the dot color intensity represents the average expression level of the indicated genes.

**b** Percentages of each cell type in the gonads of E14.5 *Kitl* f/f cre and f/+ cre female mice.

**c** Feature plots of *Kitl* and *Foxl2* in *Kitl* f/f cre and f/+ cre female mouse gonads. (0-Germ cells, 1-PreGranulosa cells, 2-PreGranulosa cells, 3-Interstitial cells, 4-Germ cells, 5-Mesothelial cells, 6-Germ cells, 7-Endothelial cells, 8-Others)

**d** Top enriched GO and KEGG terms of DEGs in granulosa cells between *Kitl* f/f cre and f/+ cre female mouse gonads.

**e** Multiviolin plot of selected meiosis-related cell marker gene expression at various stages including preleptotene, leptotene, zygotene, and pachytene. y-axis scale: a normalized UMI per-cell scale for each gene to facilitate display.

**f** Volcano plot of the differentially expressed genes in germ cells between *Kitl* f/f cre and f/+ cre female mouse gonads.

**g** qPCR of meiosis-related genes (*Meoic*, *Ythdc2*, *Spo11*) in E14.5 *Kitl* f/f cre and f/+ cre female gonads. n = 3 independent experiments.

**h** WB and quantification of differential KEGG pathways in *Kitl* f/f cre and f/+ cre female gonads. β-actin was used as a loading control. n = 2 independent experiments.

**i** GSEA of the PI3K-AKT signaling pathway and the MAPK signaling pathway in *Kitl* f/f cre and f/+ cre female gonads.

The data are presented as the means ± SDs. *p* values are derived from unpaired, two-tailed Student’s t tests (g) or two-way ANOVA (h): n.s., not significant, *p* > 0.05; * *p* < 0.05; ** *p* < 0.01; *** *p* < 0.001; **** *p* < 0.0001.


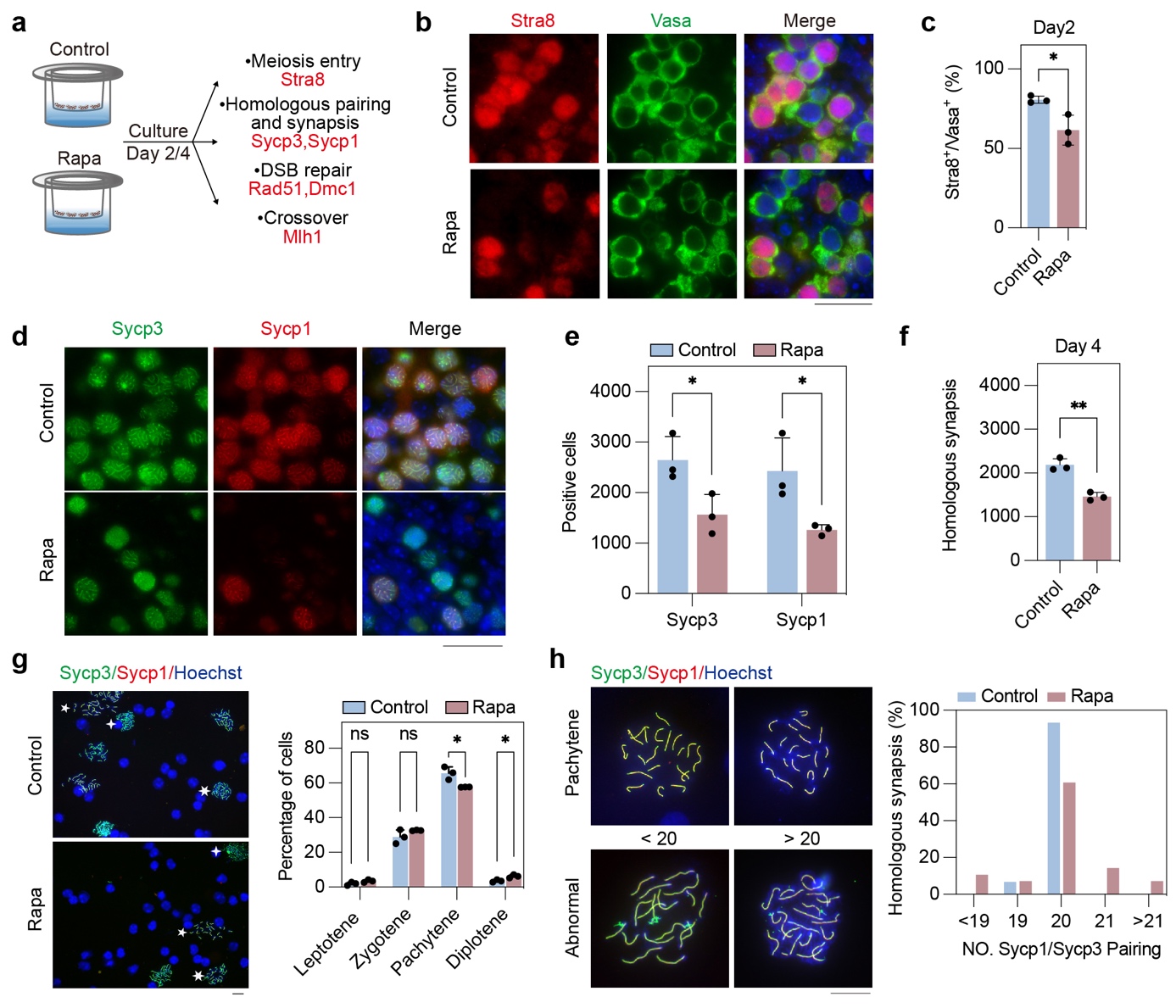


**Fig. S5 Inhibition of mTOR reduces meiosis entry and homologous synapsis.**

**a** Experimental design involving E12.5 female gonads treated with rapamycin (Rapa) or without rapamycin (control) for 2 or 4 days and examination for key meiosis markers.

**b** Immunofluorescence of Vasa and Stra8 in control and Rapa-treated gonads cultured for 2 days. Green, Vasa; red, Stra8; blue, nuclei counterstained with Hoechst 33342. Scale bar: 20 μm.

**c** Quantification of the percentage of Stra8^+^/Vasa^+^ cells in control and Rapa-treated gonads on day 2. n = 3 gonads.

**d** Immunofluorescence of Sycp1 and Sycp3 in control and Rapa-treated gonads cultured for 4 days. Green, Sycp3; red, Sycp1; blue, nuclei counterstained with Hoechst 33342. Scale bar: 20 μm.

**e** The number of Sycp3^+^ and Sycp1^+^ cells per gonad in the control and Rapa-treated gonads cultured for 4 days. n = 3 gonads.

**f** Quantification of the number of cells with homologous synapsis per gonad, as evidenced by coimmunofluorescence of Sycp1 and Sycp3 in the control and Rapa-treated gonads cultured for 4 days. n = 3 gonads.

**g** Immunofluorescence of chromosome spreads from the control and Rapa-treated gonads on day 4 stained for Sycp3 and Sycp1, and percentage of oocytes at various stages as indicated on the fourth day. Green, Sycp3; red, Sycp1; blue, nuclei counterstained with Hoechst 33342. Scale bar: 20 μm. n = 3 independent experiments.

**h** Immunofluorescence and percentage of synaptonemal complexes in normal number in the control and Rapa-treated gonads on day 4 on the basis of pachytene spread, as evidenced by co-immunofluorescence of Sycp1 and Sycp3. Scale bar: 20 μm. Scale bar: 20 μm. n = 40 nuclei for each group.

The data are presented as the means ± SDs. *p* values are derived from unpaired, two-tailed Student’s t tests (c, and f) or two-way ANOVA (e, and g): n.s., not significant, *p* > 0.05; * *p* < 0.05; ** *p* < 0.01; *** *p* < 0.001.


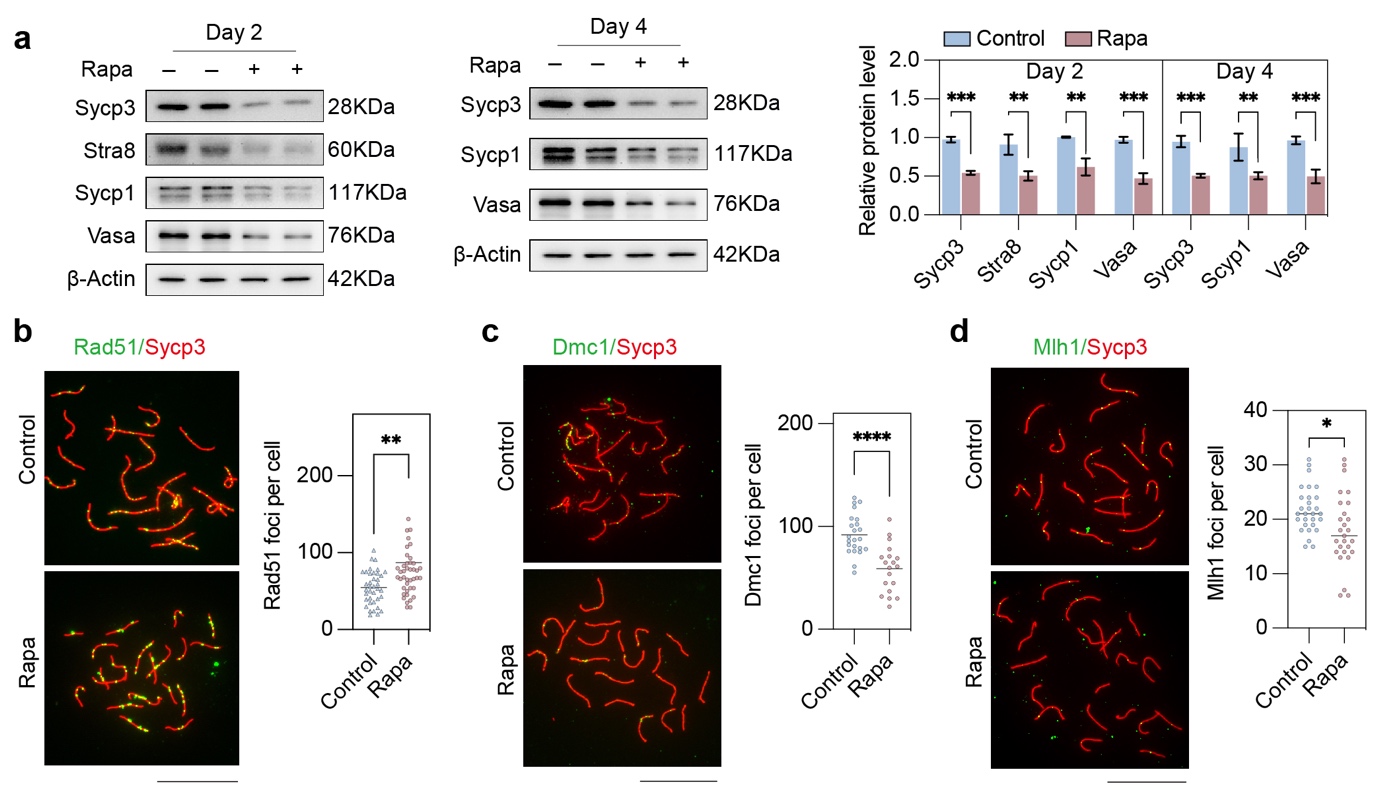


**Fig. S6 Inhibition of mTOR impairs homologous recombination.**

**a** Western blot and quantification of the Stra8, Sycp3, Sycp1 and Vasa protein levels in the control and Rapa (rapamycin, an mTOR inhibitor) groups on days 2 and 4 of culture. β-actin served as a loading control. n = 2 independent experiments.

**b** Immunofluorescence of Rad51 and Sycp3 in chromosome spreads from control and Rapa-treated gonads cultured for 4 days and quantification of the number of Rad51 foci per nucleus. Green, Rad51; red, Sycp3. n ≥ 38 nuclei for each group. Scale bar: 20 μm.

**c** Representative images of Dmc1 and Sycp3 immunofluorescence in chromosome spreads and quantification of the number of Dmc1 foci per nucleus. Green, Dmc1; red, Sycp3. n ≥ 19 nuclei for each group. Scale bar: 20 μm.

**d** Immunofluorescence of Mlh1 and Sycp3 in chromosome spreads and quantification of the number of Mlh1 foci per nucleus. Green, Mlh1; red, Sycp3. n ≥ 27 nuclei for each group. Scale bar: 20 μm.

The data are presented as the means ± SDs. *p* values are derived from unpaired, two-tailed Student’s t tests (b, c, and d) or two-way ANOVA (a): * *p* < 0.05; ** *p* < 0.01; *** *p* < 0.001; **** *p* < 0.0001.


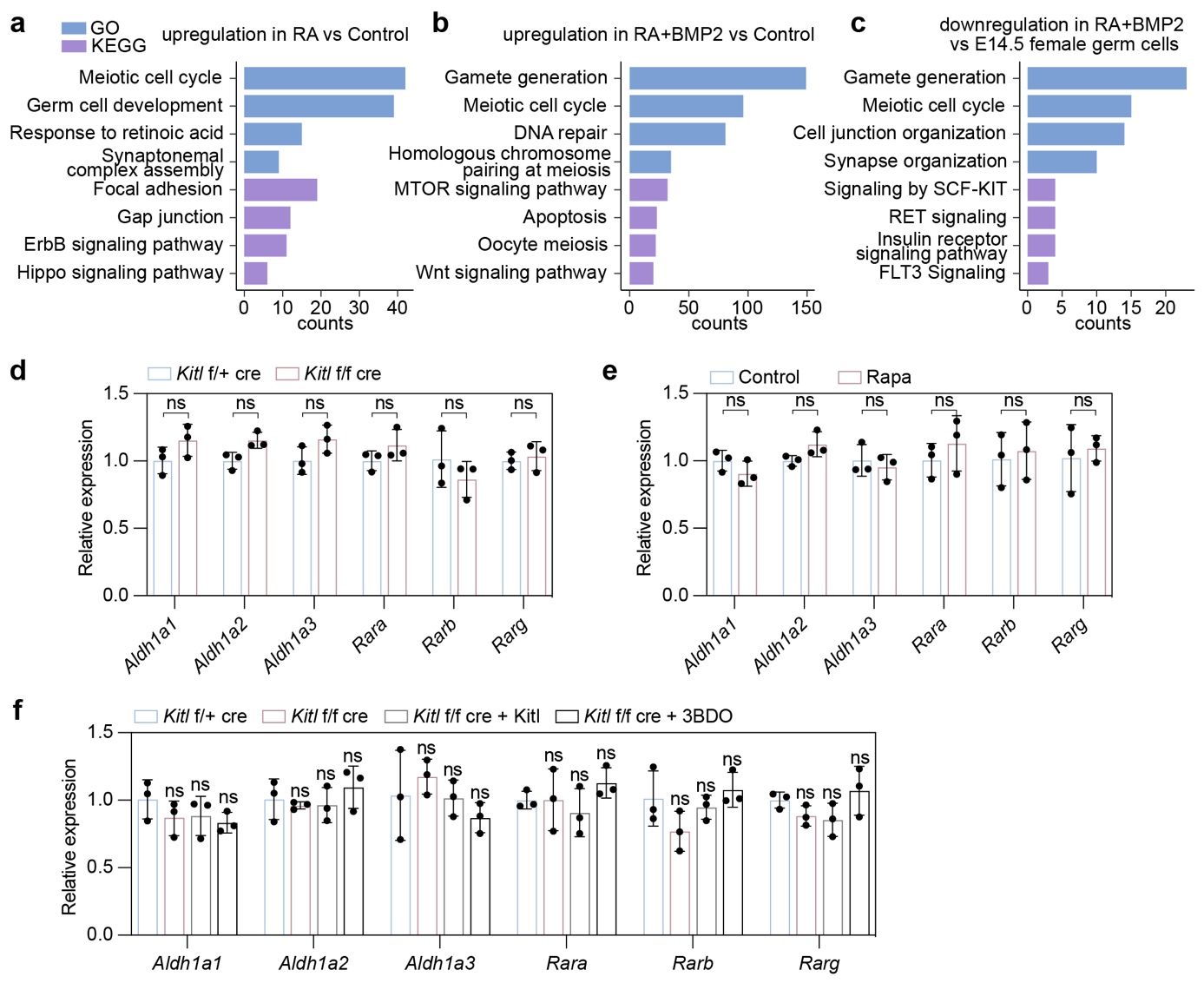


**Fig. S7 Kitl signaling acts on the meiotic entry largely independent of RA.**

**a** Upregulated genes (cutoff: FC ≥ 2, adjusted p < 0.05) in PGCLCs cultured with RA on day 9 compared with those of PGCLCs alone served as controls.

**b** Upregulated genes in PGCLCs cultured with RA and BMP2 on day 9 compared with those of PGCLCs alone served as controls on day 9.

**c** Downregulated genes in PGCLCs cultured with RA and BMP2 on day 9, compared with those of E14.5 female germ cells.

**d** qPCR analysis of RA signaling genes (*Aldh1a1*, *Aldh1a2*, *Aldh1a3*, *Rara*, *Rarb*, *Rarg*) in E14.5 *Kitl* f/f cre and f/+ cre female gonads. Data normalized to *Gapdh.*

**e** qPCR analysis of RA signaling genes in the control and Rapa-treated (Rapamycin, mTOR inhibitor) gonads cultured for 2 days.

**f** qPCR analysis of RA signaling genes in gonads of different groups include *Kitl* f/+ cre, *Kitl* f/f cre, 3BDO (mTOR activator) and Kitl on day 2 of culture.

Analysis was based on data published by Miyauchi H: GSE128553 [18].

**
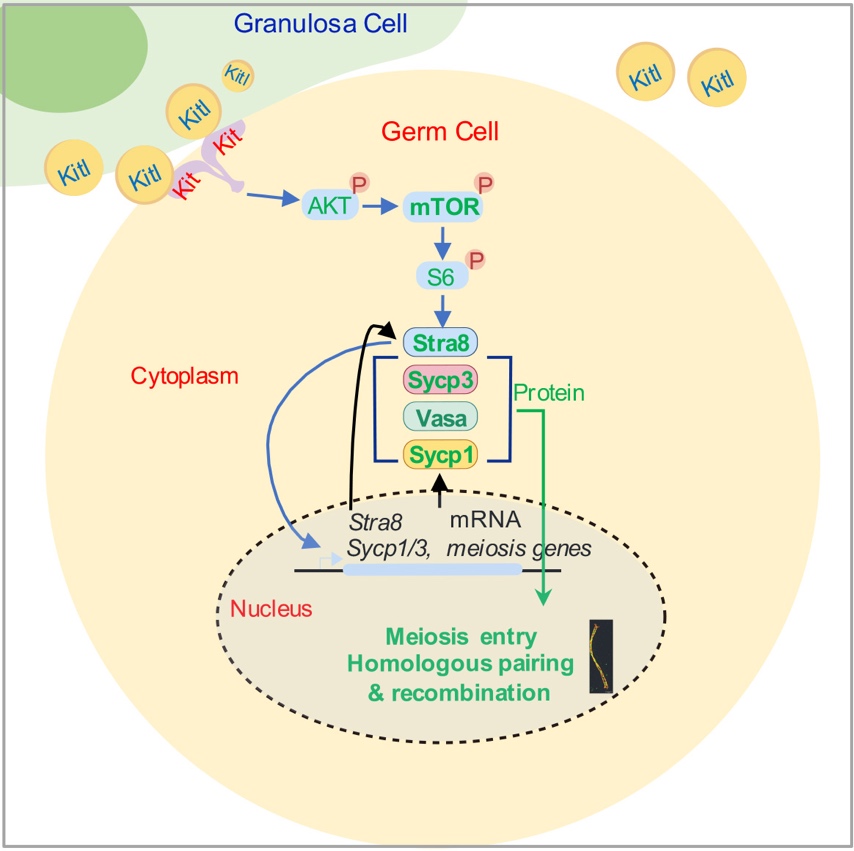
**

**Fig. S8 A simplified model displaying the Kitl/Kit/AKT/mTOR/pS6 signaling axis** **in promoting sufficient protein levels of critical genes for meiotic initiation and homologous synapsis and recombination.**

**Supplementary Table S1** **is provided separately. Genes differentially expressed between *Kitl* f/f cre and f/+ cre germ cells.**

**Supplementary Table S2 Antibody information.**

| **Protein  name** | **Manufacture (catalog number)** | **Applications** | **Website Link** |
| --- | --- | --- | --- |
|  |  | **(working dilution)** |  |
| **Vasa** | Abcam  (ab13840) | IF (1:200);  WB (1:1000) | <https://www.abcam.cn/products/primary-antibodies/ddx4--mvh-antibody-ab13840.html> |
| **Vasa** | Abcam  (ab27591) | IF (1:200) | <https://www.abcam.cn/products/primary-antibodies/ddx4--mvh-antibody-mabcam27591-ab27591.html> |
| **Sycp1** | Abcam  (ab15090) | IF (1:200);  WB (1:1000) | <https://www.abcam.cn/products/primary-antibodies/scp1-antibody-ab15090.html> |
| **Sycp3** | Abcam  (ab97672) | IF (1:200);  WB (1:1000) | <https://www.abcam.cn/products/primary-antibodies/scp3-antibody-cor-10g117-ab97672.html> |
| **Sycp3** | Novus Biologicals (NB300-230) | IF (1:200) | <https://www.novusbio.com/products/scp3-sycp3-antibody_nb300-230?keywords=NB300-230> |
| **Rad51** | Abcam  (ab133534) | IF (1:300) | <https://www.abcam.cn/products/primary-antibodies/rad51-antibody-epr40303-ab133534.html> |
| **Dmc1** | Abcam  (ab11054) | IF (1:300) | <https://www.abcam.cn/products/primary-antibodies/dmc1-antibody-2h124-ab11054.html> |
| **Stra8** | Abcam  (ab49602) | IF (1:200);  WB (1:1000) | <https://www.abcam.cn/products/primary-antibodies/stra8-antibody-ab49602.html> |
| **Kit** | R&D(AF1356) | IF (1:200);  WB (1:1000) | <https://www.rndsystems.com/cn/products/human-mouse-cd117-c-kit-antibody_af1356> |
| **Kitl** | Abcam  (ab64677) | IF (1:200);  WB (1:1000) | <https://www.abcam.cn/products/primary-antibodies/scf-antibody-ab64677.html> |
| **Foxl2** | Abcam  (ab5096) | IF (1:200) | <https://www.abcam.cn/products/primary-antibodies/foxl2-antibody-ab5096.html> |
| **Foxl2** | Abcam  (ab246511) | IF (1:200) | <https://www.abcam.cn/products/primary-antibodies/foxl2-antibody-epr23523-68-ab246511.html> |
| **Mlh1** | Proteintech  (11697-1-AP) | IF (1:100) | <https://www.ptgcn.com/products/MLH1-Antibody-11697-1-AP.htm> |
| **Cleaved**  **Caspase-3** | CST  (9961S) | IF (1:200) | https://www.cellsignal.cn/products/primary-antibodies/cleaved-caspase-3-asp175-antibody/9661 |
| **p-mTOR** | CST  (5536T) | WB (1:1000) | <https://www.cellsignal.cn/products/primary-antibodies/phospho-mtor-ser2448-d9c2-xp-rabbit-mab/5536> |
| **mTOR** | CST  (2983T) | WB (1:1000) | <https://www.cellsignal.cn/products/primary-antibodies/mtor-7c10-rabbit-mab/2983> |
| **p-S6** | CST  (4858S) | WB (1:300) | <https://www.cellsignal.cn/products/primary-antibodies/phospho-s6-ribosomal-protein-ser235-236-d57-2-2e-xp-rabbit-mab/4858> |
| **S6** | CST  (2217S) | WB (1:300) | <https://www.cellsignal.cn/products/primary-antibodies/s6-ribosomal-protein-5g10-rabbit-mab/2217> |
| **p-Stat3** | Abcam  (ab76315) | WB (1:1000) | https://www.abcam.cn/products/primary-antibodies/stat3-phospho-y705-antibody-ep2147y-ab76315.html |
| **ERK** | Santa Cruz  (SC93) | WB (1:1000) | https://www.scbt.com/zh/p/erk-1-antibody-c-16 |
| **p-ERK** | CST  (9101S) | WB (1:1000) | https://www.cellsignal.cn/products/primary-antibodies/phospho-p44-42-mapk-erk1-2-thr202-tyr204-antibody/9101 |
| **p-AKT** | CST  (2965S) | WB (1:1000) | https://www.cellsignal.cn/products/primary-antibodies/phospho-akt-thr308-c31e5e-rabbit-mab/2965 |
| **AKT** | CST  (4691S) | WB (1:1000) | https://www.cellsignal.cn/products/primary-antibodies/akt-panc67e7-rabbit-mab/4691 |
| **FoxO3a** | CST  (12829) | WB (1:1000) | https://www.cellsignal.cn/products/primary-antibodies/foxo3a-d19a7-rabbit-mab/12829 |
| **β-Actin** | ABclonal  (AC026) | WB (1:50000) | <https://abclonal.com.cn/catalog/AC026> |
| **SSEA1  magnetic beads** | Miltenyi (130-094-530) | MACS (20% of volume) | <https://www.miltenyibiotec.com/CN-en/products/anti-ssea-1-cd15-microbeads-human-and-mouse.html#130-094-530> |

**Supplementary Table S3 Chemical and recombinant protein information.**

| **Chemicals or Recombinant protein name** | **Manufacture (catalog number)** | **working concentration** | **Website Link** |
| --- | --- | --- | --- |
| **ISCK03** | MCE  (HY-101443) | 10μM | https://www.medchemexpress.cn/isck03.html?src=bd-product |
| **Kitl** | PeproTech  (250-03) | 100 ng/mL | https://www.thermofisher.cn/cn/zh/proteins/product/Mouse-SCF-Recombinant-Protein/250-03-100UG |
| **3BDO** | MCE  (HY-U00434) | 10μM | https://www.medchemexpress.cn/3BDO.html |
| **VC** | Sigma  (A8960) | 50μg/mL | https://www.sigmaaldrich.cn/CN/en/product/sigma/a8960 |
| **RA** | Sigma  (R2625) | 100 nM | https://www.sigmaaldrich.cn/CN/en/product/sigma/r2625 |
| **BMP2** | PeproTech  (120-02-100) | 300 ng/mL | https://www.thermofisher.cn/cn/zh/proteins/product/Human-Mouse-Rat-BMP-2-Recombinant-Protein/120-02-10UG |
| **SC79** | MCE  (HY-18749) | 10μM | https://www.medchemexpress.cn/SC79.html?src=bd-product |
| **Rapamycin** | LC Laboratories  (R-5000) | 0.5μM | https://www.lclabs.com/ |
| **Y-27632 2HCl (Rocki)** | Selleck  (S1049) | 10μM | https://www.selleck.cn/products/Y-27632.html |

**Supplementary Table S4 Primer sequences.**

| **Primer** | **Gene target** | **Application** | **Sequences (5′-3′)** |
| --- | --- | --- | --- |
| *Foxl2*-Cre-Forward | *Foxl2* | genotype | CGGCATGGTGCAAGTTGAAT |
| *Foxl2*-Cre-Reverse | *Foxl2* | genotype | TCAGCTACACCAGAGACGGA |
| *Kitl*-flox-Forward | *Kitl* | genotype | CGAGGTAGGGGAAAAGAACC |
| *Kitl*-flox-Reverse | *Kitl* | genotype | GGATCTTCCCAGAGGTTGGA |
| *Spo11*-qpcr-Forward | *Spo11* | qRT‒PCR | AGCATGAAGTGTCTCACTAGCA |
| *Spo11*-qpcr-Reverse | *Spo11* | qRT‒PCR | CATTAACAGGGCAAGGCACCTA |
| *Meioc*-qpcr-Forward | *Meioc* | qRT‒PCR | AATCTTGGTGCCTAAGTCTATG |
| *Meioc*-qpcr-Reverse | *Meioc* | qRT‒PCR | AGGCTTTATATCCAGCAACTC |
| *Ythdc2*-qpcr-Forward | *Ythdc2* | qRT‒PCR | GGTCCGATCAATCATCTGT |
| *Ythdc2*-qpcr-Reverse | *Ythdc2* | qRT‒PCR | GAAGTAACGAATAGGCATGT |
| *Stra8*-qpcr-Forward | *Stra8* | qRT‒PCR | GCCGGAGAAGGAGGAGATTAAA |
| *Stra8*-qpcr-Reverse | *Stra8* | qRT‒PCR | AGCAGCCTTTCTCAATGAGTCT |
| *Aldh1a1*-qpcr-Forward | *Aldh1a1* | qRT‒PCR | ACTTTCCCACCATTGAGTGC |
| *Aldh1a1*-qpcr- Reverse | *Aldh1a1* | qRT‒PCR | CACCATGGATGCTTCAGAGA |
| *Aldh1a2*-qpcr-Forward | *Aldh1a2* | qRT‒PCR | CATGGTATCCTCCGCAATG |
| *Aldh1a2*-qpcr- Reverse | *Aldh1a2* | qRT‒PCR | GCGCATTTAAGGCATTGTAAC |
| *Aldh1a3*-qpcr-Forward | *Aldh1a3* | qRT‒PCR | TCTGGGAATGGCAGAGAACT |
| *Aldh1a3*-qpcr- Reverse | *Aldh1a3* | qRT‒PCR | TTGATGGTGACGGTTTTCAC |
| *Rara*-qpcr-Forward | *Rara* | qRT‒PCR | AAATCATCCGGCTACCACT |
| *Rara*-qpcr- Reverse | *Rara* | qRT‒PCR | TCTGGATGCTTCGTCGGAA |
| *Rarb*-qpcr-Forward | *Rarb* | qRT‒PCR | GTGTTCACCTTTGCCAACCAG |
| *Rarb*-qpcr- Reverse | *Rarb* | qRT‒PCR | TTTAGTGCTTCCAGCAGTGGT |
| *Rarg*-qpcr-Forward | *Rarg* | qRT‒PCR | TCTTCTGGCTACCACTATGGGGTCA |
| *Rarg*-qpcr- Reverse | *Rarg* | qRT‒PCR | GCAGTACTGGCATCGATTTCTGG |
| *mGapdh*-qpcr- Forward | *Gapdh* | qRT‒PCR | AGGTCGGTGTGAACGGATTTG |
| *mGapdh*-qpcr-Reverse | *Gapdh* | qRT‒PCR | TGTAGACCATGTAGTTGAGGTCA |

**Supplementary Table S5 Summary of Statistical Test Results.**

| **Figure** | **Normality test**  **(Shapiro-Wilk test)** | **homogeneity of variance test**  **(Levene's test)** | **Test method** | **Significance** |
| --- | --- | --- | --- | --- |
| **Fig. 2d** | No | Yes | Mann-Whitney U test | 0.0470 (*) |
| **Fig. 2f**  **Vasa** | Yes | Yes | t-test | 0.0341 (*) |
| **Fig. 2f**  **Sycp3** | Yes | Yes | t-test | 0.0477 (*) |
| **Fig. 2f**  **Sycp1** | Yes | Yes | t-test | 0.0122 (*) |
| **Fig. 2g** | Yes | Yes | t-test | 0.0442 (*) |
| **Fig. 2i**  **Vasa** | Yes | Yes | t-test | 0.0453 (*) |
| **Fig. 2i**  **Sycp3** | Yes | Yes | t-test | 0.0198 (*) |
| **Fig. 2i**  **Sycp1** | Yes | Yes | t-test | 0.0263 (*) |
| **Fig. 2j** | Yes | Yes | t-test | 0.0311 (*) |
| **Fig. 3c**  **Lep** | Yes | Yes | t-test | 0.2352 (ns) |
| **Fig. 3c**  **Zyg** | Yes | Yes | t-test | 0.0138 (*) |
| **Fig. 3c**  **Pac** | Yes | Yes | t-test | 0.0015 (**) |
| **Fig. 3c**  **Dip** | Yes | Yes | t-test | 0.6307 (ns) |
| **Fig. 3d** | No | Yes | Mann-Whitney U test | 0.0114 (*) |
| **Fig. 3f** | No | No | Mann-Whitney U test | 0.0151 (*) |
| **Fig. 3g** | Yes | Yes | t-test | <0.0001  (****) |
| **Fig. 3h** | No | No | Mann-Whitney U test | <0.0001  (****) |
| **Fig. 5c** | Yes | Yes | t-test | 0.0020 (**) |
| **Fig. 5e**  **Sycp3** | Yes | Yes | t-test | 0.0116 (*) |
| **Fig. 5e**  **Sycp1** | No | Yes | Mann-Whitney U test | 0.0109 (*) |
| **Fig. 5f** | Yes | Yes | t-test | 0.0227 (*) |
| **Fig. 5h**  **Lep** | Yes | Yes | t-test | 0.7408 (ns) |
| **Fig. 5h**  **Zyg** | Yes | Yes | t-test | 0.0316 (*) |
| **Fig. 5h**  **Pac** | Yes | Yes | t-test | 0.0044 (**) |
| **Fig. 5h**  **Dip** | Yes | Yes | t-test | 0.3811 (ns) |
| **Fig. 5j** | No | Yes | Mann-Whitney U test | 0.0011 (**) |
| **Fig. 5k** | No | No | Mann-Whitney U test | <0.0001  (****) |
| **Fig. 5l** | No | Yes | Mann-Whitney U test | 0.0005 (***) |
| **Fig. 6c** | No | Yes | Kruskal-Wallis test | As shown in Fig. 6c |
| **Fig. 6d** | No | Yes | Kruskal-Wallis test | As shown in Fig. 6d |
| **Fig. 6h** | Yes | Yes | One-way ANOVA | As shown in Fig. 6h |
| **Fig. 6i** | Yes | Yes | One-way ANOVA | As shown in Fig. 6i |
| **Fig. 6j** | Yes | Yes | One-way ANOVA | As shown in Fig. 6j |
| **Fig. 8d** | Yes | Yes | One-way ANOVA | As shown in Fig. 8d |
| **Fig. 8f**  **Stra8** | No | No | Kruskal-Wallis test | As shown in Fig. 8f |
| **Fig. 8f**  **Sycp1** | No | No | Kruskal-Wallis test | As shown in Fig. 8f |
| **Fig. 8f**  **Sycp3** | No | No | Kruskal-Wallis test | As shown in Fig. 8f |
| **Fig. S5c** | Yes | Yes | t-test | 0.0265 (*) |
| **Fig. S5e**  **Sycp3** | Yes | Yes | t-test | 0.0369 (*) |
| **Fig. S5e**  **Sycp1** | Yes | Yes | t-test | 0.0378 (*) |
| **Fig. S5f** | Yes | Yes | t-test | 0.0019 (**) |
| **Fig. S5g**  **Lep** | Yes | Yes | t-test | 0.1109 (ns) |
| **Fig. S5g**  **Zyg** | Yes | Yes | t-test | 0.1841(ns) |
| **Fig. S5g**  **Pac** | Yes | Yes | t-test | 0.0204 (*) |
| **Fig. S5g**  **Dip** | Yes | Yes | t-test | 0.0177 (*) |
| **Fig. S6b** | No | Yes | Mann-Whitney U test | 0.0016 (**) |
| **Fig. S6c** | Yes | Yes | t-test | <0.0001  (****) |
| **Fig. S6d** | Yes | Yes | t-test | 0.0133 (*) |
