# Supplementary figures and images for: Somatic Kitl promotes mTOR to facilitate prophase I of meiosis in female embryonic gonads
Source: Cell Death Dis. 2025 Nov 17;16(1):838. doi: 10.1038/s41419-025-08158-y (PMC12623929; doi:10.1038/s41419-025-08158-y)

Fig.2k

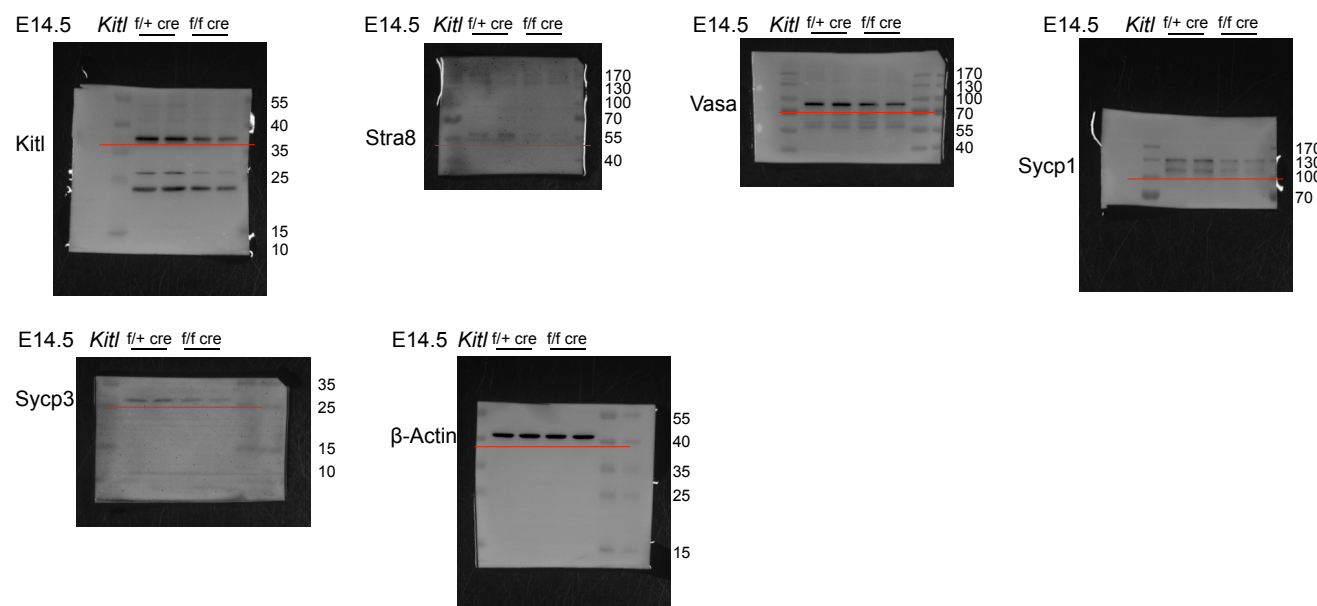

Fig.2l

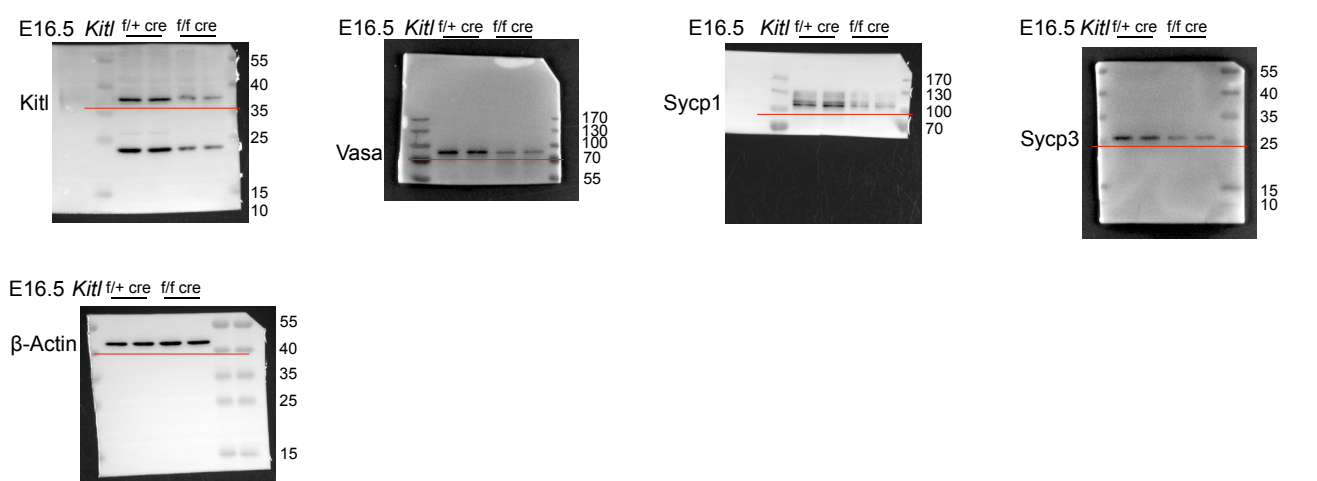

Fig.5i

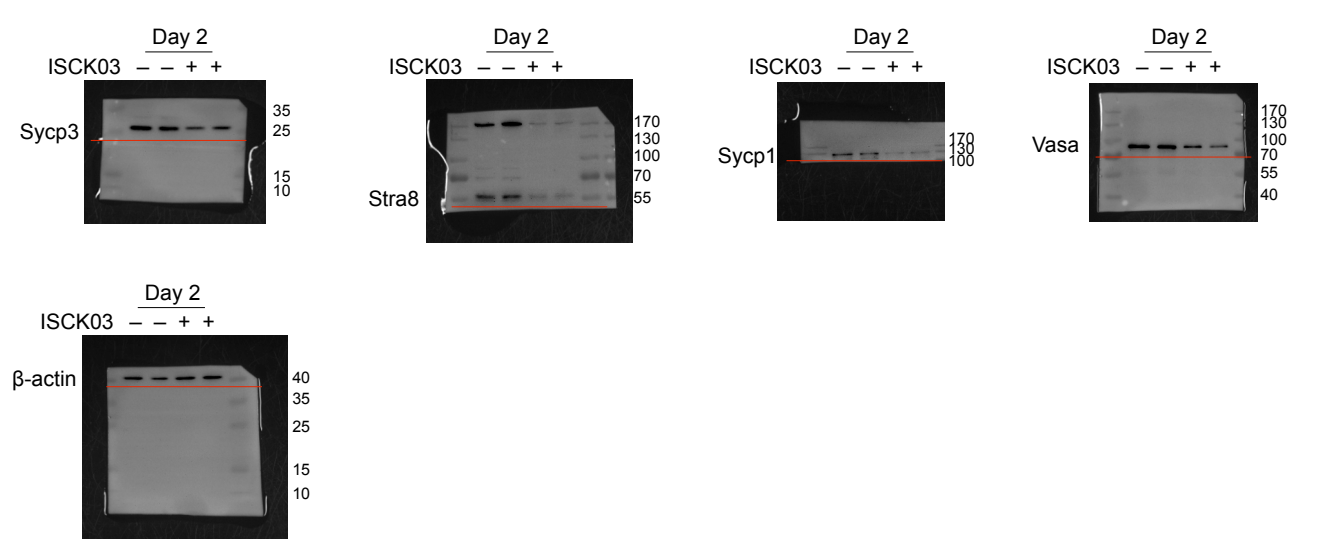

Fig.5i

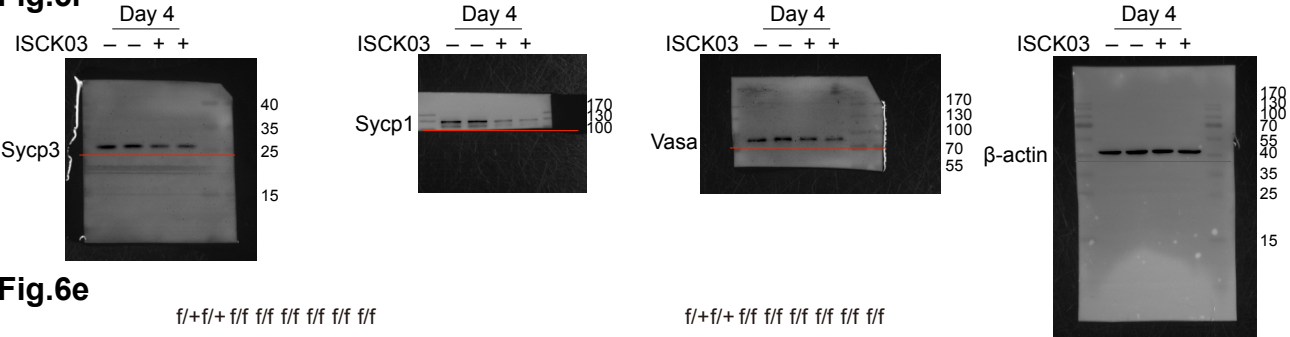

Fig.6e

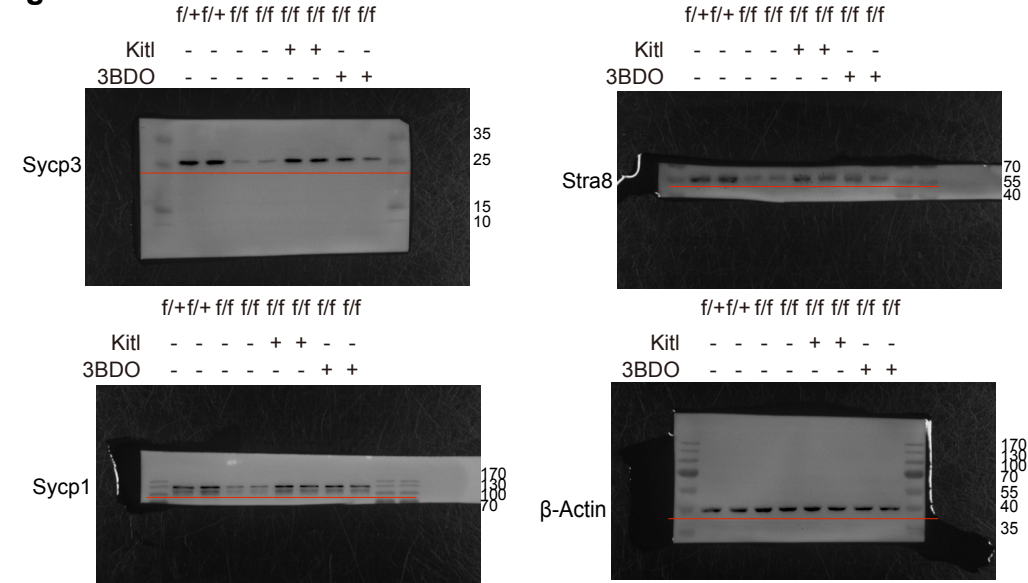

Fig.7a

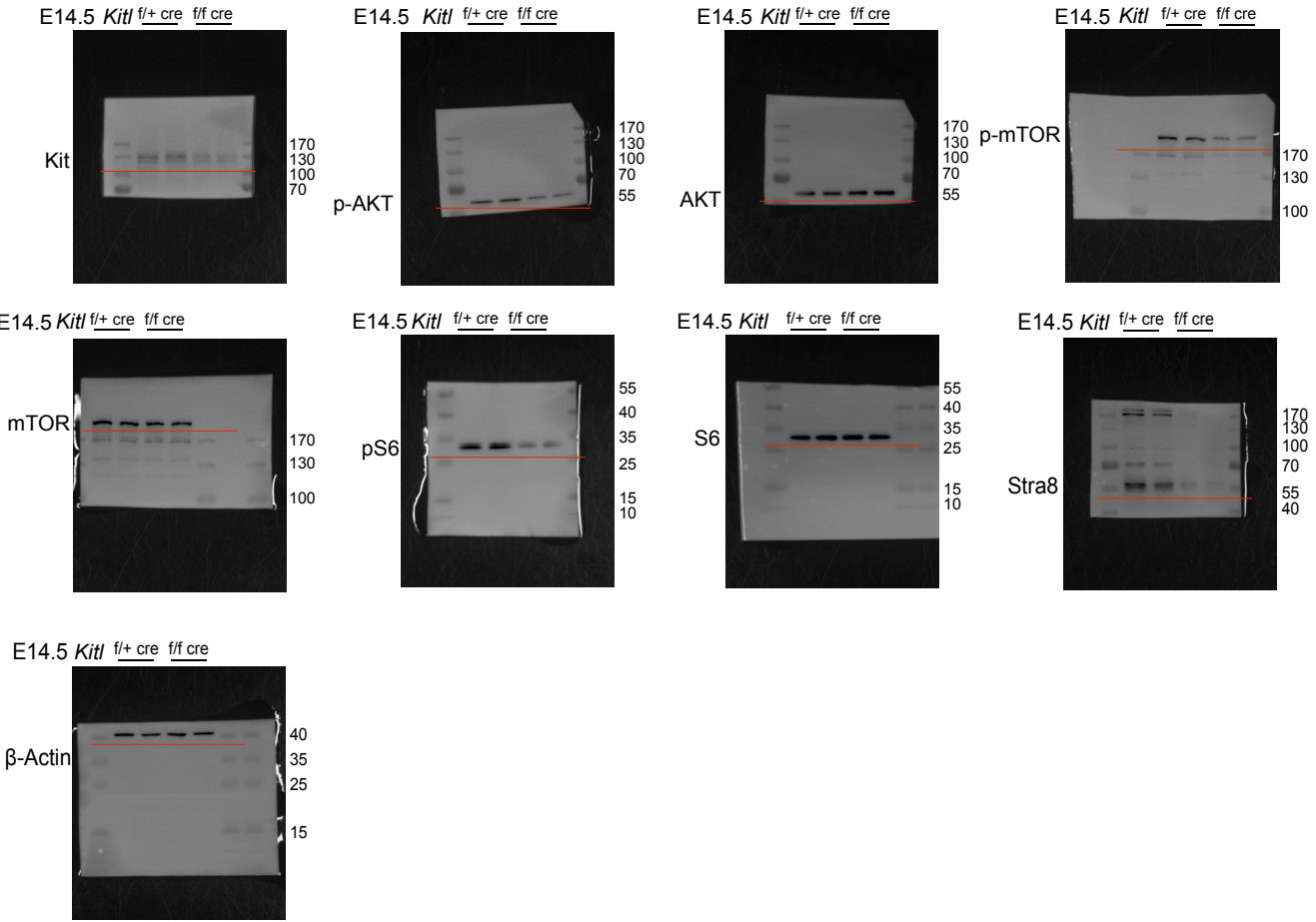

**Fig.7b**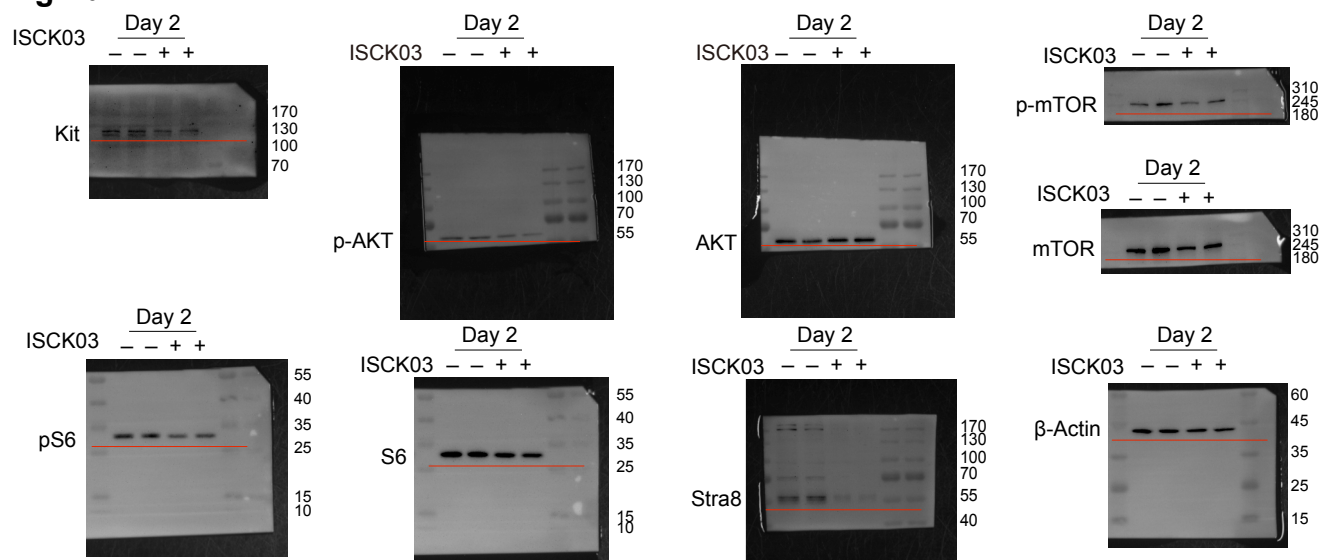**Fig.7c**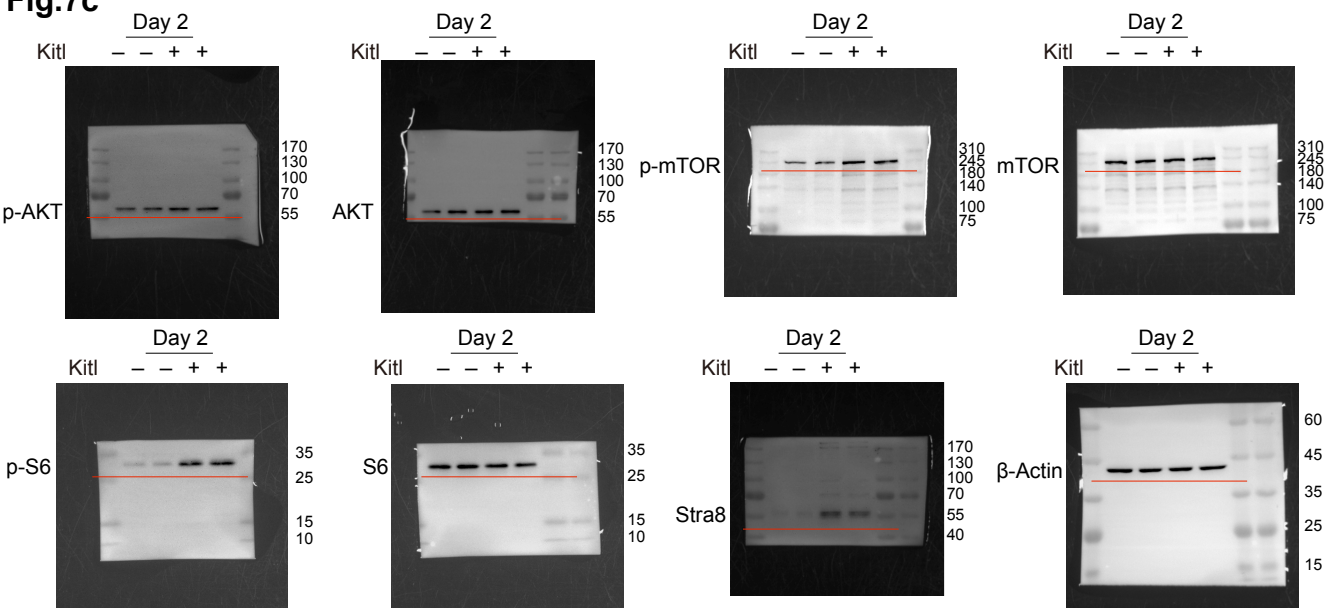**Fig.7d**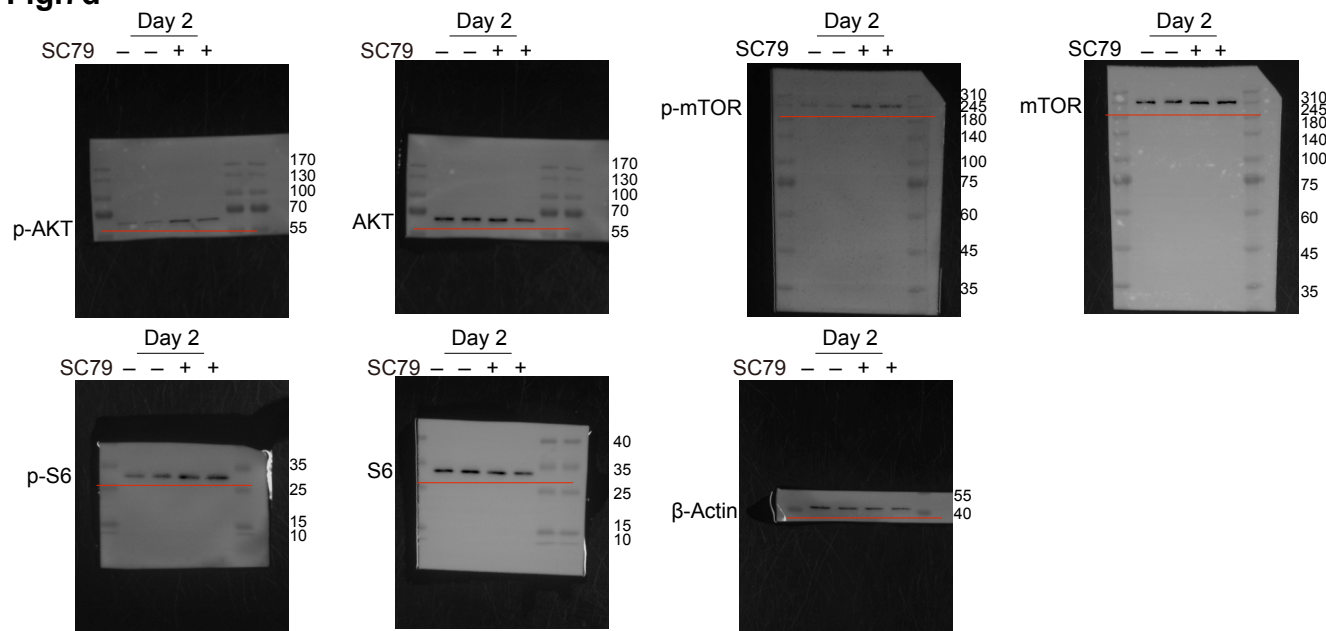

Fig.7e

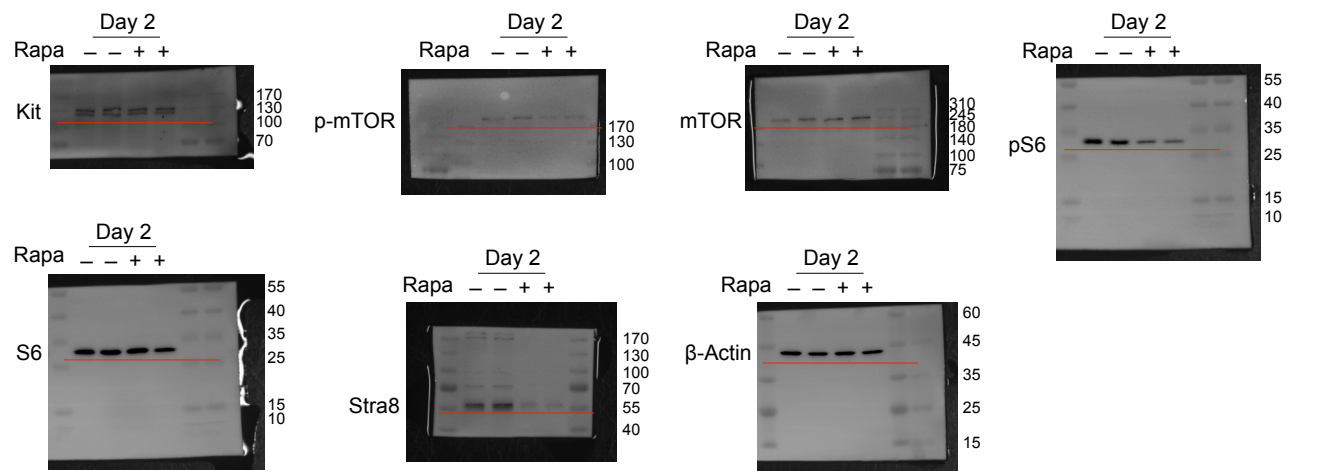

Fig.7f

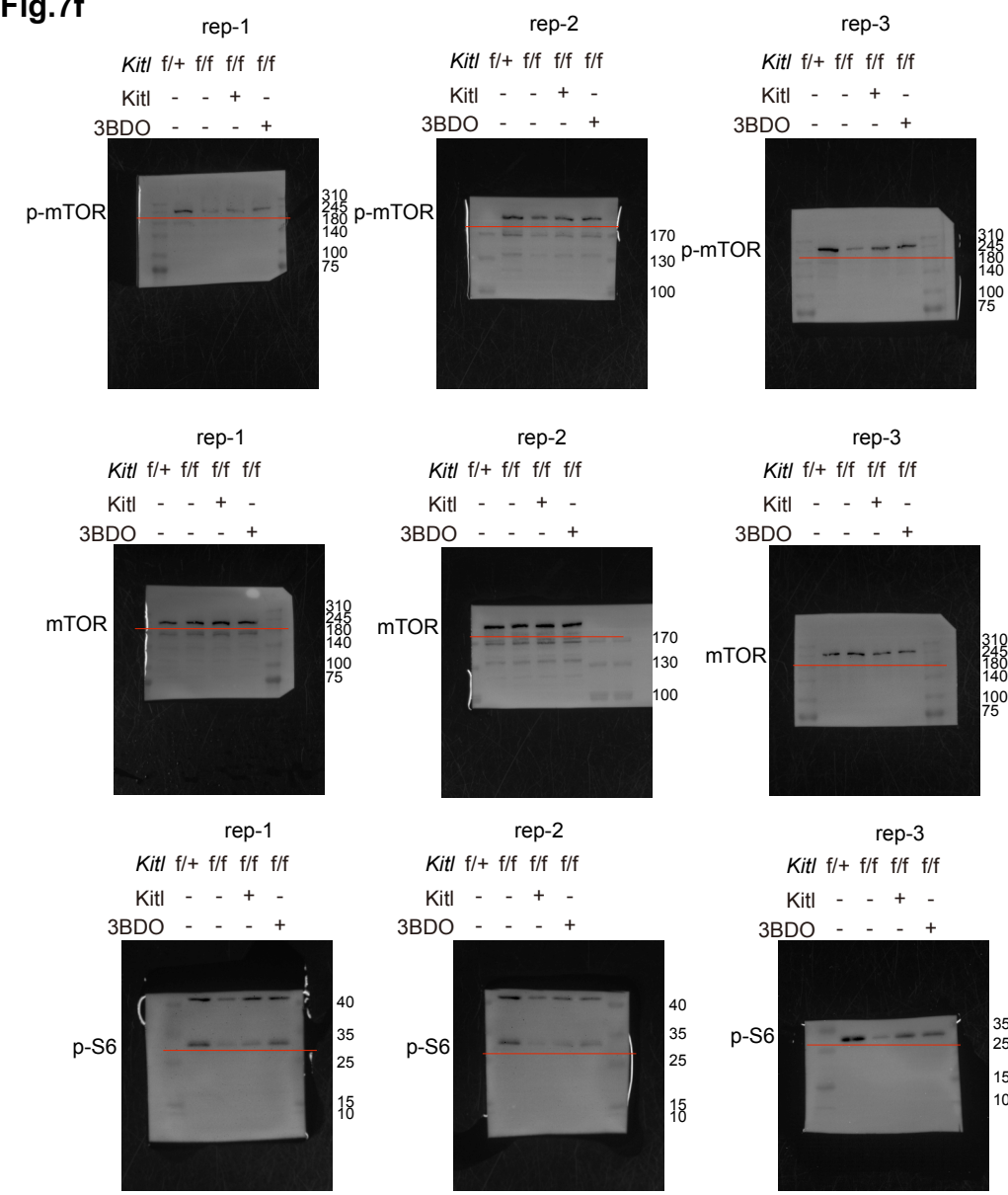



Fig.S3h

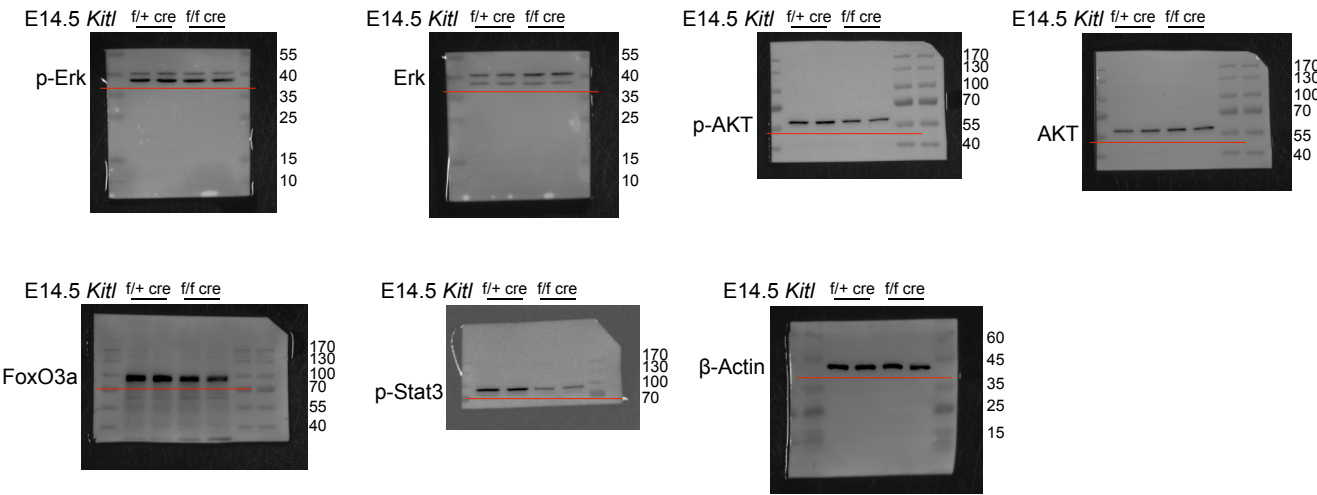

Fig.S5a

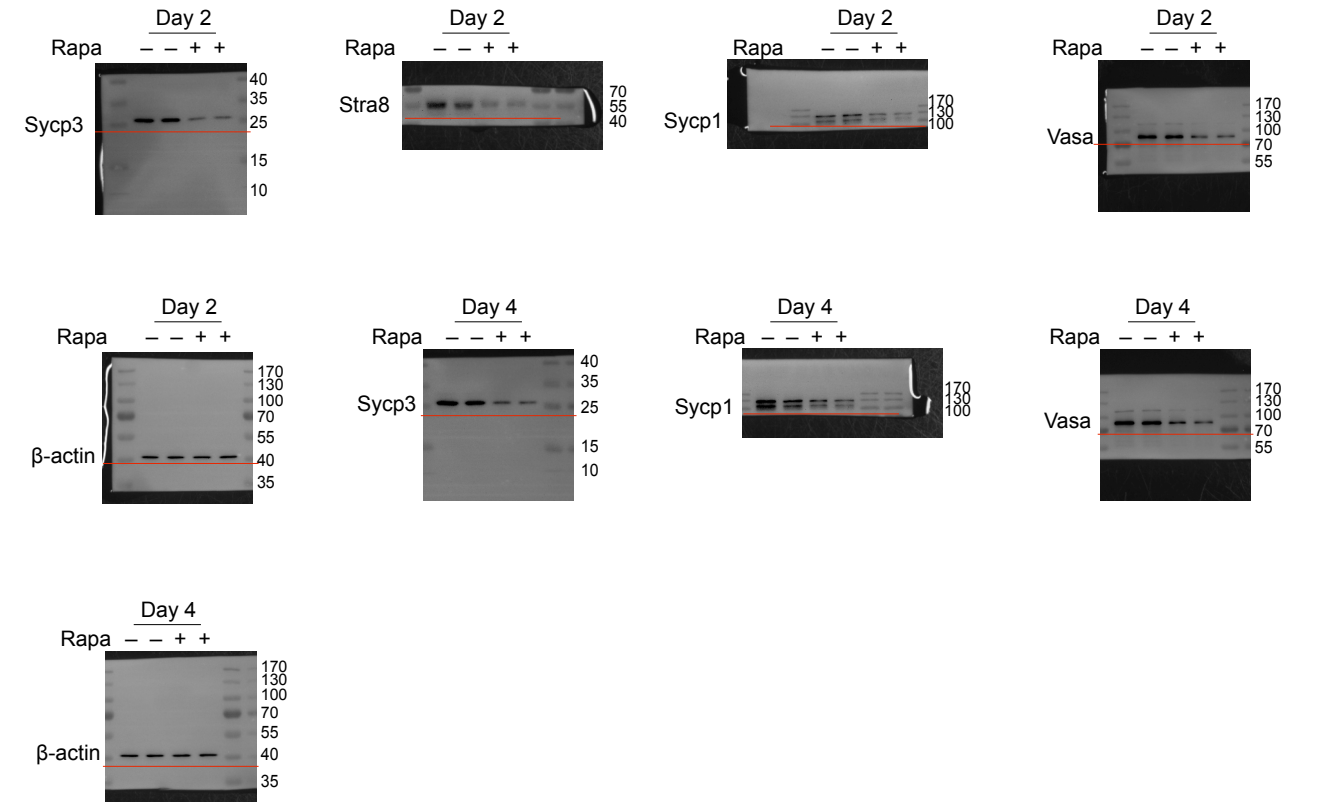

Supplement: Supplementary file 4 — Original western blots [file 41419_2025_8158_MOESM4_ESM.pdf]
